# Low Energy Subsurface Environments as Extraterrestrial Analogs

**DOI:** 10.3389/fmicb.2018.01605

**Published:** 2018-07-18

**Authors:** Rose M. Jones, Jacqueline M. Goordial, Beth N. Orcutt

**Affiliations:** Bigelow Laboratory for Ocean Sciences, East Boothbay, ME, United States

**Keywords:** deep biosphere, subsurface, astrobiology, low energy, energy limitation

## Abstract

Earth’s subsurface is often isolated from phototrophic energy sources and characterized by chemotrophic modes of life. These environments are often oligotrophic and limited in electron donors or electron acceptors, and include continental crust, subseafloor oceanic crust, and marine sediment as well as subglacial lakes and the subsurface of polar desert soils. These low energy subsurface environments are therefore uniquely positioned for examining minimum energetic requirements and adaptations for chemotrophic life. Current targets for astrobiology investigations of extant life are planetary bodies with largely inhospitable surfaces, such as Mars, Europa, and Enceladus. Subsurface environments on Earth thus serve as analogs to explore possibilities of subsurface life on extraterrestrial bodies. The purpose of this review is to provide an overview of subsurface environments as potential analogs, and the features of microbial communities existing in these low energy environments, with particular emphasis on how they inform the study of energetic limits required for life. The thermodynamic energetic calculations presented here suggest that free energy yields of reactions and energy density of some metabolic redox reactions on Mars, Europa, Enceladus, and Titan could be comparable to analog environments in Earth’s low energy subsurface habitats.

## Introduction

### Astrobiology and Life Under Energy Limitation

**FIGURE 1:**
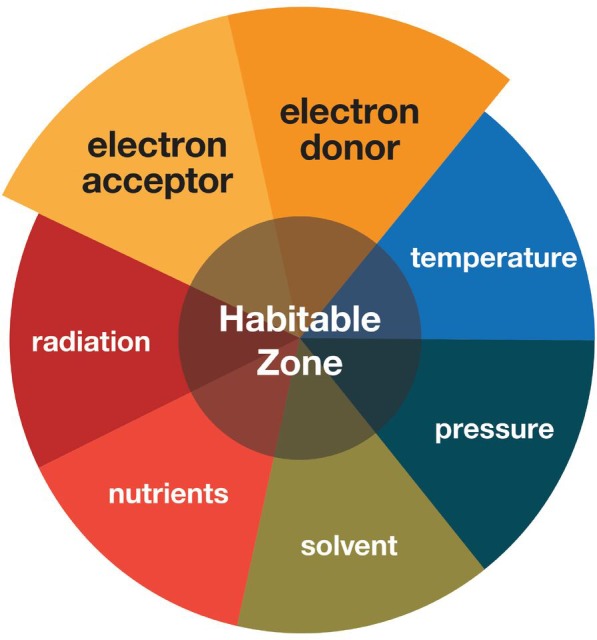
Schematic illustrating the different parameters that contribute to habitability. This review primarily focuses on the electron donor and electron acceptor parameters on extraterrestrial targets and Earth’s low energy subsurface environments.

Astrobiology includes the search for the presence of life outside the Earth (Domagal-Goldman et al., 2016). The immensity of this challenge requires a focused search, which involves setting constraints on where life may and may not be possible. Setting the boundaries of this habitable zone in a meaningful way (i.e., neither too broad nor too limiting) is not trivial and can be defined by a number of parameters (**Figure 1**), each with its own advantages and limitations that compete and complement each other (Cockell et al., 2016).

Analog sites on Earth are those that share past or present characteristics with other planetary bodies, providing natural systems for study of the limits of life, which are often quite different from lab conditions (Arndt et al., 2013). This concept is based on the idea that laws of physics and chemistry are universal, a principle that underlies a large proportion of astrobiology research (Léveillé, 2010; Preston and Dartnell, 2014). Therefore, sites on Earth can provide information on how physical and chemical conditions interact to form environments conducive to life elsewhere (Lederberg, 1960; Léveillé, 2010). “Extreme” environments [where conditions fall outside of the “standard” of 4–40°C, pH 5–8.5, and salinity above 37 g kg^−1^ water (Kristjánsson and Hreggvidsson, 1995; Bartlett and Bidle, 1999)] are common analog targets used as a way to identify and develop tools to search for identifying signs that life is or was ever present under a range of conditions (Preston and Dartnell, 2014). The current primary targets for astrobiology investigations within the solar system are Mars, Enceladus, Europa, and possibly Titan. These planetary bodies have surface conditions that are largely considered inhospitable to life, but where subsurface conditions are potentially habitable.

To better understand life under low energy conditions for extrapolation to extraterrestrial targets, studying environments on Earth that experience limited energetic disequilibria is useful. This “follow the energy” approach to evaluating whether life is possible relies on the idea that chemical disequilibria are important, providing differences in potential energy that can be used to drive reactions required by life (Kappler et al., 2005; Barge and White, 2017). The difference between equilibrium states of a given redox couple (i.e., an electron donor and an electron acceptor) defines this disequilibrium and how much energy could be released, with the equilibrium tipping point dependent on state variables like temperature and pressure. A gradient of redox pairs is generated; those of highest yield are generally removed preferentially and redox pairs of least energy yield persist. This corresponds to an approximate trend for decreasing microbial activity (Zhang et al., 2016), particularly in low disturbance environments such as sediments. Thus, defining the habitable zone (**Figure 1**) requires identifying electron donor/acceptor pairs that supply sufficient potential energy to satisfy the energetic limits of life under realistic state variables.

The energetic limit of life is the minimum energy (i.e., difference in redox potential) necessary for a cell. Electron transport in a cell works either by having each protein along the path at a slightly lower redox potential than the previous protein to facilitate movement of electrons from one to the next (Anraku, 1988) or by having an ion concentration gradient across the membrane to drive ATP production (Müller and Hess, 2017). Pathways and energetic requirements vary between genus and metabolism, with some microbes able to use multiple transport pathways (Kracke et al., 2015; Lever et al., 2015; Müller and Hess, 2017). Physical constraints such as resources required to perform and maintain particular metabolic reactions have an effect on which redox reactions are metabolically favorable (Amenabar et al., 2017).

The purpose of this review is to provide an overview of Earth’s low energy subsurface sites as potential analog environments with particular emphasis on how they inform the study of the energetic limits required for life to exist, which has implications for refining the search for extraterrestrial life. These resource limits have applications in defining the energetic aspect of habitability, including minimum thresholds and identification of possible electron acceptor/donor reactions. This review compliments other recent reviews of astrobiology and analogs (Domagal-Goldman et al., 2016; Martins et al., 2017), habitability (Cockell et al., 2016), energetics and astrobiology (Barge and White, 2017), forward contamination (Fairén et al., 2017), deep marine environments (Fisher, 2005; Orcutt et al., 2013a), deep continental environments (Fredrickson and Balkwill, 2006; Colman et al., 2017), and energy within these (Amend and Teske, 2005; Edwards et al., 2012a; Hoehler and Jørgensen, 2013; Bach, 2016; Bradley et al., 2018). After reviewing basic features of Earth’s subsurface environments and extraterrestrial targets, we review current understanding of energy limitation for life, and conclude with new assessments of possible chemotrophic energy availability in the subsurface analogs and extraterrestrial sites.

### Defining the Low Energy Subsurface

**FIGURE 2:**
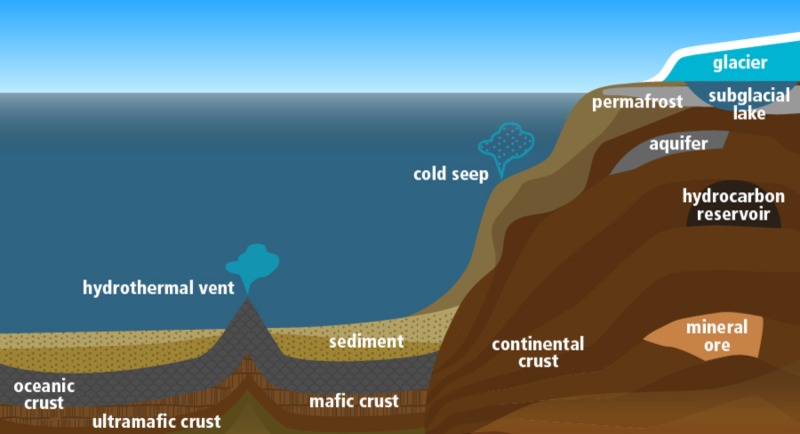
Cross-section schematic of low energy subsurface environments on Earth.

The subsurface begins below the solid surface of the earth and includes a wide range of conditions across microscopic and macroscopic scales, substrate age and accumulation rates. The subsurface is classified into various continental and marine environments (**Figure 2**). These terms are rarely explicitly defined but usually refer to whether there is land or ocean above a location (Whitman et al., 1998; McMahon and Parnell, 2014), or to whether a location is situated in a continental or marine tectonic plate (Cogley, 1984). The continental definition generally includes continental shelves as continental, whereas the former will class them as marine. Note that the term “terrestrial” is often used in place of “continental” in the literature; however, we avoid use of the term terrestrial in this context, as this term encompasses the entire Earth system [i.e., “intraterrestrial” life (Edwards et al., 2012a) and “extraterrestrial” life].

Current definitions of the boundaries of the subsurface are somewhat imprecise, and yet highlight why the subsurface is relevant as a source of analog sites. Classifications of the shallowest boundary of the “deep subsurface” have included depth (Jørgensen and Boetius, 2007; Edwards et al., 2012a), pressure (Oger and Jebbar, 2010), water flow (Lovley and Chapelle, 1995), and operational considerations (Orcutt et al., 2011). These thresholds generally follow the principle that surface processes influence shallow sites while deep subsurface sites are more isolated (i.e., do not interact with surface products and processes) and less prone to disturbance. However, this definition does not hold up when considering all environments within continental and marine environments, such as subglacial lakes and desert varnishes. Likewise, the downward extent to which life penetrates is poorly constrained. The upper temperature limit for life is generally taken as the ultimate constraint for the lower depth limit (Takai et al., 2008), and likely varies between environments due to the influence of other physical and chemical characteristics (Wilhelms et al., 2001; Head et al., 2003; LaRowe et al., 2017). The working definition used here (as in Edwards et al., 2012a; Wu et al., 2016; Colman et al., 2017) is of a low energy subsurface environment with all requirements for life are sourced from surrounding substrate. As such, the environments considered herein come from a variety of depths as we considered the threshold at which this definition becomes a true function of local conditions. For example, using our definition, sites with slow deposition rates and little disturbance such as oligotrophic sediment and Antarctic permafrost may have a subsurface that begins only a few cm below the actual surface. We also considered the overlaying surface immaterial. Therefore, ice covered sites are included within our definition.

Some characteristics are common to subsurface environments of Earth. Light is mostly absent (Van Dover et al., 1996; Reynolds and Lutz, 2001; White et al., 2002; Beatty et al., 2005), generally eliminating phototrophy as a lifestyle. Chemical-based lithotrophic reactions support a large fraction of life in Earth’s subsurface environments, though transport of organic matter and oxidants of photosynthetic origin (i.e., oxygen) introduces influences from Earth’s surface. In comparison to surface environments, deep subsurface sites on Earth are often limited by low concentrations of electron donors, electron acceptors, carbon and/or nutrients (D’Hondt et al., 2009, 2015; Hoehler and Jørgensen, 2013; Lever et al., 2015). Subsurface environments often have limited permeability and/or porosity, restricting transport and motility and experience higher pressures compared to the surface world, in addition to occasional extremes in pH and elevated temperature (Lysnes et al., 2004; Kelley et al., 2005; Prokofeva et al., 2005; Slobodkin and Slobodkina, 2014).

## Earth’s Subsurface Habitat Types

### Marine

Beginning beneath the seafloor, there are three major habitat types: marine sediment, oceanic crust, and seep environments (**Figure 2**). Boundaries between these provide gradients and thresholds that provide additional opportunities for life.

#### Marine Sediment

The marine sediment subsurface begins below the bioturbation zone, where burrowing animals actively disturb the sediment. Bioturbation depth varies depending on the rate of sedimentation of particles and the organic content of the particles (Jørgensen and Boetius, 2007; Teal et al., 2008). Generally, sedimentation rate and organic content load is highest near continental margins where particles and nutrients are shed from land, and lowest beneath the center of ocean gyres. Marine sediment composition varies from sands to fine-grained clays, and from biologically derived oozes to continentally derived particles and volcanic ashes. Porosity of marine sediment, and consequently the volume of liquid within it, decreases with depth due to compaction, and recent estimates suggest that roughly 5% of Earth’s water is in the form of pore water within sediment (LaRowe et al., 2017). In locations with significant organic carbon burial, typically on continental shelves, methane production from terminal electron accepting processes can lead to the formation of large quantities of dissolved and free methane gas. Some of this gas escapes the seafloor, supporting “cold seep” environments where methane oxidation supports chemotrophic communities, with the majority of the gas stored as gas hydrates or clathrate ices (Whiticar, 1990; Clennell et al., 1999; Koh, 2002; Katayama et al., 2016).

There is vertical zonation of chemotrophic biogeochemical processes in marine sediment as more energy-rich terminal electron acceptors are preferentially used to oxidize organic carbon, generally in the order of dissolved oxygen, nitrate, metal oxides, sulfate, and carbon dioxide, followed by fermentation (Froelich et al., 1979; Orcutt et al., 2011; Arndt et al., 2013). Labile organic carbon is used first, with less favorable fractions persisting for hundreds to millions of years (Arndt et al., 2013). In areas with little organic carbon delivery, like beneath ocean gyres, the rate of organic carbon oxidation is so slow that relatively energy rich electron acceptors (i.e., oxygen, nitrate) penetrate throughout the entire sediment column (D’Hondt et al., 2009, 2015; Orcutt et al., 2013b). These low-resource sediments are not necessarily extremely limited across all metabolisms, however, as rates of activity for particular metabolic reactions are comparable to those of less oligotrophic sediments (Orcutt et al., 2013a). Growth and persistence rates in the order of <1–∼350 years are proposed in such oligotrophic sediments (Biddle et al., 2006; Braun et al., 2017; Volpi et al., 2017), reflecting metabolic limitations caused by scarcity of electron donors in addition to nutrients. In such conditions, it is necessary to consider dormancy and maintenance (Orcutt et al., 2013a; Lever et al., 2015; Reese et al., 2018). Yet the latest biomass estimates in sediment are ∼2.9 × 10^29^ cells or ∼0.6% of earth’s total biomass (Kallmeyer et al., 2012), indicating that it is possible for communities to persist in these environments.

#### Oceanic Crust

The oceanic crust begins beneath sediment cover or as exposed seafloor where ocean crust is newly formed or not blanketed by sediment. There are two main lithologies in the oceanic crust: an upper mafic layer of extrusive and intrusive basalts above intrusive gabbros, and a deeper ultramafic layer (Karson, 2002). Mafic rocks are generally low in silica (55–45 weight percent) and have a relatively high iron and magnesium oxide content. Ultramafic rocks have less silica (<45 weight percent) and more iron and magnesium. In contrast to sediments, the ocean crust is generally scarce in organic carbon and nitrogen-poor and includes increased trace elements depending on the host geology (Staudigel et al., 1998).

The oceanic crust encompasses not just the host rocks, but the fluids that circulate through them (Furnes and Staudigel, 1999; Edwards et al., 2005; Orcutt et al., 2011). Ocean water enters exposed areas of crust and moves through cracks and fissures in the crust, exiting as diffuse or focused flow due to pressure and temperature changes driving siphon effects (Fisher, 2005). Circulation depth is poorly constrained, extending >500 m below the crustal surface (Furnes and Staudigel, 1999). Fluid volume in the oceanic crust is estimated at ∼2% of the total ocean volume (Edwards et al., 2005) and circulates the entire ocean volume equivalent every ∼10^5^–10^6^ years (Becker and Fisher, 2000). It is a complex habitat, with interactions between rocks (primarily iron-silicates such as basalt) and fluids that vary in temperature (cool to several hundreds of degrees Celsius), redox state (oxic to highly reducing), and pH (acidic to basic). Variation over time occurs with continual creation of freshly exposed rock surfaces due to volcanic eruptions or tectonic events and closing off of flow pathways due to alteration or deformation. Faster rates of subseafloor flow could therefore result in a more diverse, larger, and active community as more resources are delivered and inhibitors removed per unit of time compared to a system with a slower rate (Zhang et al., 2016). Where this altered fluid seeps into the sediment above, it introduces new sources of electron acceptors and donors (Engelen et al., 2008; Ziebis et al., 2012; Orcutt et al., 2013b; Labonté et al., 2017).

It was only recently appreciated that large portions of oceanic crust are oxic, due to seafloor hydrothermal circulation replenishing oxygen at depth and limited drawdown of oxygen in oligotrophic sediment above (Røy et al., 2012; Ziebis et al., 2012; Arndt et al., 2013; Orcutt et al., 2013b; D’Hondt et al., 2015; Braun et al., 2017). Chemolithotrophic electron donors and acceptors such as oxidized and reduced sulfur, iron, and manganese compounds are common in the crust (Bach and Edwards, 2003; Edwards et al., 2012a). The majority of these metal oxides are in solid form under near-neutral to alkaline subsurface conditions, but there are microbes that directly transfer electrons directly from the mineral for use in energy pathways (Lovley, 2008; Smith et al., 2014; Badalamenti et al., 2016). Hydrogen may be an important electron source, particularly in the lower layers (Amend and Shock, 2001; Bach, 2016), though the generation mechanism is as yet unclear (Chapelle et al., 2002; Brazelton et al., 2013; Dzaugis et al., 2016). Less is known about the ocean crustal subsurface in general, partially due to the operational challenges of sampling this environment. Crustal estimates of biomass and rates of activity are currently poorly constrained, though energetic calculation suggest this environment could support up to ∼1 × 10^12^ g C yr^−1^ of new biomass (Bach and Edwards, 2003; Edwards et al., 2005), and recent empirical measurement of chemotrophic carbon fixation support this (Orcutt et al., 2015).

#### Seep Habitats

The marine environment has two types of seep habitats: cold seeps (described above) and hydrothermal seeps (commonly referred to as hydrothermal vents). Though not strictly deep subsurface environments, these environments provide natural “windows” into otherwise challenging to access environments and are therefore considered. Fluids in these environments are subject to interactions with the local geology, resulting in an altered physiochemical character such as enrichment with dissolved minerals and metals (Staudigel et al., 1998). Marine hydrothermal vent systems represent concentrated regions of chemotrophic life in the otherwise oligotrophic deep-sea floor, as warm, mineral rich subseafloor fluids mix with cold, oxic, resource-deficient surface waters. This enrichment provides a potentially energy rich environment, particularly where reduced chemical species mix with oxic seawater in plume environments (Dick et al., 2013).

### Continental

The continental subsurface begins beneath the top active layers of soil and ice or directly under exposed crust (**Figure 2**). There are three main habitat types in the continental subsurface: the crust, ores (here including hydrocarbon deposits) and aquifers. There are also “windows into the subsurface” environments such as hydrothermal springs and ophiolites.

#### Continental Crust

The continental crust has an estimated volume of approximately 2 × 10^8^ km^2^ or 40% of the Earth’s solid volume, depending on the definition (Cogley, 1984). It is primarily granitic (high concentration of silica) and highly heterogeneous, becoming mafic at depth (Wedepohl, 1995; Rudnick and Gao, 2003). This contrasts with the broadly homogeneous marine crust which has a low silica content (Rudnick and Fountain, 1995). This heterogeneity provides a wide variety of habitats for chemolithotrophs, as there is more substrate variability in addition to temperature and pressure gradients. “Window” sections of the deepest subsurface are accessible as uplifted ophiolites (Neubeck et al., 2017; Rempfert et al., 2017). These environments provide information on deeper geology and interactions between these and microbial communities, though the degree to which these sites are reflective of the true deep subsurface varies according to local conditions.

Water in the terrestrial subsurface is primarily in channels and pore spaces. The solid to water ratio tends to increase with depth, causing a more constrained environment further down (Stober and Bucher, 2004; Parnell and McMahon, 2016). Water becomes more saline at depth, as long residence times of thousands of years results in the accumulation of dissolved minerals from contact with rock. Total groundwater volume in the terrestrial subsurface crust is estimated at 2.43 × 10^19^ L (Wirsen and Jannasch, 1978), with an estimated max volume of 98% in aquifers (Gleeson et al., 2016). These aquifers are areas of more permeable rock and sediment where groundwater has less restricted flow (Foster and Chilton, 2003). Aquifer conditions vary according to surrounding geology and sometimes anthropogenic effects, but are in general oligotrophic.

Similar to the marine crust, electron acceptors and donors include iron (Emerson et al., 2007; Heim et al., 2017), manganese (Peng et al., 2015; Sylvan et al., 2015), sulfur species and hydrogen (Osburn et al., 2014). Use of other metal species is reported but their significance is often unclear (Kashefi and Lovley, 2000; Oremland, 2003; Lee et al., 2015). Clays and sediments are occasionally present, which can have a higher organic carbon content than rock crust (Bagnoud et al., 2016). Elevated concentrations of hydrogen in particular are present in the deeper subsurface due to radiolysis, or as a product of serpentinization, a geochemical alteration process that produces methane, hydrogen, and abiotically produced hydrocarbons as a result of water-ultramafic rock interactions. Similar to the deep crustal marine subsurface, estimated rates of activity in the continental deep subsurface are poorly constrained, due in part to difficulties and costs involved in collecting quality samples from deep boreholes and mine systems (Miettinen et al., 2015; Momper et al., 2017).

#### Ores and Mineral Deposits

Concentrations of a particular mineral can alter local conditions and microbiology (Lehman et al., 2001), though they are rarely of significant volume in comparison to the total crustal volume (Williams and Cloete, 2008; Daly et al., 2016). Ores are mineral concentrations of economic interest, and here includes hydrocarbon (e.g., coal) and halite deposits in addition to metal-containing ores. These ores are sometimes the subject of commercial exploitation, which provides opportunities for site access though mining activity (Osburn et al., 2014; Miettinen et al., 2015; Daly et al., 2016). However, these environments are by definition altered by anthropogenic activity, changing local conditions, and local microbiology. Examples include generation of acid mine drainage (Druschel et al., 2004; Chen et al., 2016), souring of hydrocarbon deposits (Head et al., 2014), and alterations by explosive activity (Martins et al., 2017).

The range of electron acceptors and donors in these environments can be very different from typical crust. Hydrocarbon deposits such as oil and coal contain abundant organic carbon, though this is usually not labile and often includes toxic aromatic compounds (Larter et al., 2006; Head et al., 2014). Depending on host geology, sulfate in particular provides a reasonably energy rich electron acceptor (Sánchez-Andrea et al., 2011; Dopson and Johnson, 2012; Tsesmetzis et al., 2016). Other metals such as manganese (Spilde et al., 2005), arsenic (Escudero et al., 2013), uranium (Beller et al., 2013), and others (Johnson et al., 2017) are used in addition to iron as electron sources and sinks (Hedrich et al., 2011; Toner et al., 2016). Metal species in particular are usually present in low dissolved concentrations in the environment regardless of their concentration in solid mineral because of their respective solubility under environmental conditions. There are exceptions such as low pH environments associated with some ores and hydrothermal fluids, where a high concentration of H^+^ shifts the thermodynamics of solid/liquid/gas state equilibrium, changing solubility so more metals stay in solution (Hedrich et al., 2011; Johnson et al., 2012). There is evidence that iron, sulfur, and methane oxidizing microbes use hydrogen as an electron donor in these environments (Lau et al., 2016; Carere et al., 2017; Hernsdorf et al., 2017).

#### Cold Subsurface Environments

Cold, hyperarid deserts are some of the closest analogs to current extraterrestrial targets for life, such as high elevation McMurdo Dry Valleys in Antarctica (Heldmann et al., 2013) and the Atacama Desert in Chile (Navarro-González et al., 2003; Fairén et al., 2010). Temperatures in these environments rarely reach above freezing, resulting in a soil profile almost exclusively of permafrosts. These are oligotrophic systems, with potential electron acceptors such as nitrates, sulfates, and perchlorates as well as electron donors such as formate, acetate, and other small organic acids are present in these primarily mineral soils (Kounaves et al., 2010b; Parro et al., 2011; Jackson et al., 2016; Faucher et al., 2017).

Subglacial lakes such as those in Greenland, Iceland, and Antarctica (Siegert et al., 2016) are considered as ‘subsurface’ herein, as they fit the definition of an environment relatively isolated from surface processes. These are bodies of water of varying fluid dynamics, physiochemical properties, and residence times that may or may not have a rocky bottom, capped by kilometers of ice (Bell et al., 2002; Mikucki et al., 2016; Siegert et al., 2016). Microbial studies of these sub-glacial lakes show lithotrophic communities with unique microbial members and metabolisms with a range of electron donors and acceptors, with less biomass associates with the higher ice-water boundaries (Murray et al., 2012; Bulat, 2016; Mikucki et al., 2016).

## Extraterrestrial Astrobiological Targets with Subsurface Analogs on Earth

Mars, Europa, Enceladus, and Titan have received the most attention as the most likely to harbor signs of past or present extraterrestrial life. These sites possess characteristics or specific sites that share similar aspects of particular low energy subsurface sites, in terms of physical characteristics and possible energy sources.

### Mars

Present day Mars is cold and hyper arid with low atmospheric pressure (∼7 mbar), high ionizing radiation, and highly oxidizing surface soil conditions (Fairén et al., 2010). Surface conditions on Mars are currently considered inhospitable because of this and due to the instability of liquid water on the surface. Water is present on Mars in the near subsurface in the form of ground ice and potentially as ground water residing in deeper crust (Clifford and Parker, 2001; Byrne et al., 2009; Clifford et al., 2010). Subsurface conditions such as pressure above the triple point of water, radiogenic heating and the presence of dissolved solutes could allow for liquid water with depth (Clifford et al., 2010). Evidence indicates that past Mars was relatively warmer than at present and liquid water was widespread (Fairén et al., 2010), resulting in possible previously habitable conditions. There are microorganisms on Earth that grow at subzero temperatures (Mykytczuk et al., 2013) and under present Martian atmosphere conditions (Mickol and Kral, 2017). Extant life could potentially remain in the more clement subsurface conditions due to protection provided from ionizing radiation and surface soil oxidizing conditions.

Basalts, clays and ultramafic minerals are found across Mars, and provide possible lithotrophic electron donors and acceptor sources. Iron and sulfur in particular are abundant (Squyres et al., 2004; Nixon et al., 2013), and there is evidence of perchlorates (Catling et al., 2010; Navarro-González et al., 2010), nitrogen (Stern et al., 2015), and other metal-containing minerals (Squyres et al., 2004). Oxygen is present in the atmosphere in extremely low concentrations of 0.1%, likely due to abiotic formation (Kounaves et al., 2010a). Evidence that Martian conditions have been locally extremely acidic (Horgan et al., 2017; Peretyazhko et al., 2017), means that metal and sulfur ions in particular could have been more bioavailable similar to low pH environments on Earth (Fernández-Remolar et al., 2008; Amils et al., 2014). Serpentine deposits have been identified associated with impact craters and surface terrains by the Mars Reconnaissance Orbiter in a number of sites on Mars (Ehlmann et al., 2010), including Nili Fossae, a site possibly linked with enriched methane concentrations (Mumma et al., 2009). Since methane is thought to have a short lifespan in the Martian atmosphere, a potential source for the variable detection of methane on Mars (Webster et al., 2015) could be present-day and/or past serpentinization processes in the subsurface (Oehler and Etiope, 2017) as relevant minerals are present (Hoefen et al., 2003; Ody et al., 2012). Some have theorized that the Martian subsurface could be supplied with an energy source from the oxidation of photochemically produced H_2_ and CO diffusing into regolith, penetrating down to 100–1,000 meters (Weiss et al., 2000). Microorganisms in surface cold and hyper-arid soils are identified that utilize these substrates as a carbon and energy source in trace atmospheric amounts (Ji et al., 2017), and CO oxidation has been identified as a metabolism in the subsurface on Earth (Brazelton et al., 2012; Baker et al., 2016; Hoshino and Inagaki, 2017).

Analog environments to the cold and hyper-arid conditions on Mars include the subsurface of the hyperarid Atacama desert in Chile as well as polar deserts (Navarro-González et al., 2003; Fairén et al., 2010). While the dry surface mineral soils of the Atacama desert harbor little to no active microbial life (Navarro-González et al., 2003; Crits-Christoph et al., 2013), there is a “microbial oasis” at depth (∼2 m), where small films of liquid water is formed due to deliquescence caused by hygroscopic salts (Parro et al., 2011). In Antarctica, the McMurdo Dry Valleys are a polar desert analog similar to observations at the *Phoenix* landing site, where dry permafrost soil with negligible water content overlays ice-cemented ground (Heldmann et al., 2013). Life is likely constrained more by available liquid water than low energy in these oligotrophic environments (Goordial et al., 2016) as active microbial life in these cold, dry valleys can be observed at lower elevations where liquid water is more prevalent seasonally (Bakermans et al., 2014).

Considering the abundance of basalts and ultramafics on Mars, the subsurface provides analogs for Martian minerals, including basalts, clays, and serpentine (Stevens and Mckinley, 1995; Schulte et al., 2006) among others. Serpentinite hosted microbial ecosystems are found in subsurface environments in marine (e.g., Atlantis Massif) and in terrestrial settings (e.g., the Tablelands Ophiolite, The Ceders) (Kelley et al., 2005; Brazelton et al., 2006; Hernsdorf et al., 2017; Rempfert et al., 2017). Ophiolites on earth are identified as analogs for similar lithologies on Mars as a source of hydrogen in particular, and depending on the host substrate, other energy sources such as iron or manganese may also be present (Schulte et al., 2006; Szponar et al., 2013; Dilek and Furnes, 2014). Deeply occurring clays (Bagnoud et al., 2016; Leupin et al., 2017) and particularly oligotrophic marine sediment (D’Hondt et al., 2009) may be useful analogs for Martian organic-poor clays.

### Icy Moons With Liquid Oceans

There are currently two primary icy ocean world targets for possible extraterrestrial life: Saturn’s moon Enceladus, and Europa, a moon of Jupiter. Enceladus probably has a rocky core covered at least partially by an approximately 10 km deep body of probably alkaline (pH 8.0–12: Postberg et al., 2009; Glein et al., 2015; Hsu et al., 2015) brine (Vance et al., 2016), with a cap of 30–40 km of ice (Iess et al., 2014). There is a geyser in the southern hemisphere (the so-called ‘tiger stripes’), speculated to be sourced from hydrothermal activity (Glein et al., 2015; Hsu et al., 2015; Sekine et al., 2015) or clathrate decomposition (Kieffer et al., 2006). Information collected from the geyser indicate the presence of carbon, nitrogen and organic compounds (CH_4_, NH_4_), silicates (Postberg et al., 2009; Waite et al., 2009), sodium, potassium and carbonates (Postberg et al., 2009) and moderate salinity (Glein et al., 2015; Hsu et al., 2015). Temperature estimates for subsurface ocean range from <90°C in the assumed crustal subsurface to approximately 0°C near the ice-water interface. There are indications of possibly significant water-rock interactions between the liquid body and a hypothesized solid rock core (Glein et al., 2015; Hsu et al., 2015).

Europa is hypothesized to contain a rocky basaltic core (Vance et al., 2016) in contact with a liquid brine ocean ∼80–100 km deep (Kivelson et al., 2000; Lowell and DuBose, 2005), with a ∼15–25 km shell of ice (Kivelson et al., 2000) and lakes encased within (Schmidt et al., 2011). Conditions of pH, temperature, and composition of brines in Europa are less constrained than Enceladus. though the presence of H_2_O_2_, O_2_, SO_2_, CO_2_, carbonates, and sulfates are inferred from spectral data of surface ice (McCord et al., 1998; Carlson et al., 1999; Hand et al., 2007), which are theorized to enter the subsurface in some instances (Teolis et al., 2017). Proposed possible metabolisms include methanogenesis and sulfate reduction pathways (McCollom, 1999), though evidence of compounds involved in these pathways have yet to be detected in the surface ice (Hand et al., 2007). If oxygen enters the subsurface, then this may also function as an electron acceptor, though the actual concentrations involved may be low and localized (Teolis et al., 2017). Hydrothermal activity and subsurface flow, are speculated based on features of the ice surface (Lowell and DuBose, 2005; Quick and Marsh, 2016).

Considering the ultramafic, saline, and alkaline conditions hypothesized to exist on these icy moon targets, marine ultramafic subsurface sites such as Lost City on the Atlantis Massif are useful to consider as they are broadly similar (Postberg et al., 2009; Preston and Dartnell, 2014; Glein et al., 2015; Vance et al., 2016; Lunine, 2017). The Lost City vent system consists of a succession of alkaline (pH 9–11) vents of ∼45–90°C fluids. The Europa site at the Mid-Cayman Ridge is another cool, ultramafic-hosted system with elevated methane (German et al., 2010). In these sites, seawater is entrained within the ultramafic system and undergoes fluid-rock reactions that remove carbonate, adds alkalinity, and enriches hydrogen, methane, and other small weight organics that are byproducts of serpentinization reactions. Serpentinization systems such as these have been speculated as a possible energy source on Enceladaus (Glein et al., 2015; Holm et al., 2015), with a possible H_2_ generation value of approximately 3 mol H_2_ kg^−1^ of water (Glein et al., 2015). This compares to 0.25–15 mmol H_2_ kg^−1^ of water in the Lost City hydrothermal vent system (Kelley et al., 2001, 2005; Proskurowski et al., 2008). Methane and sulfates are a possible electron donor and acceptor (Kelley et al., 2001; Lang et al., 2010; Brazelton et al., 2013; Schrenk et al., 2013); however, a recent study suggests sulfate-reducers dominate this environment despite abundant methane (Lang et al., 2018). There is little sulfate yet detected on Enceladus, so sulfur couples may not be a significant source of energy on this target (Glein et al., 2015).

Basaltic oceanic crust environments also serve as useful analogs to icy ocean worlds, given the interaction of saline fluids with crust inferred on these targets. The best-studied subsurface environments on Earth where fluid moves through oceanic crust are on the flanks of mid-ocean ridges, namely the eastern flank of the Juan de Fuca Ridge and the western flank of the Mid-Atlantic Ridge at the “North Pond” site (Edwards et al., 2012b; Orcutt and Edwards, 2014). Recent studies at these sites have revealed dynamic microbial ecosystems thriving in this subsurface environment (Jungbluth et al., 2016; Meyer et al., 2016; Tully et al., 2018).

Considering the ice-water interface on these icy moons, proposed analogs for ice-water interfaces include sea ice-water channels (Martin and McMinn, 2017) and sub-glacial lakes such as Lake Vida, an Antarctic permanently ice-covered brine lake (Murray et al., 2012; Garcia-Lopez and Cid, 2017). Sub-glacial lake studies show chemotrophic communities with unique community members and evidence of more life at substrate-water interfaces than ice-water interface, indicating life is possible in these conditions (Murray et al., 2012; Bulat, 2016; Mikucki et al., 2016). Identified metabolisms include sulfate reduction, methanogenesis, nitrate as an electron acceptor (Skidmore et al., 2000; Michaud et al., 2017) and, at the crust-water interface, lithotrophic sources such as manganese and iron (Murray et al., 2012).

Considering the presence of brines in contact with crust on icy moons, salt ores and cold seep brine pools offer additional subsurface analogs for icy moons. Brine pools with distinct microbial communities are present in the marine subsurface, forming as buried salt deposits interact with upwelling subsurface ocean fluids (Joye et al., 2009, 2010; Antunes et al., 2011). Deep terrestrial halite ores harbor microbial communities, including inclusions within the deposits (Lowenstein et al., 2011; Jaakkola et al., 2016; Payler et al., 2017). Ionic strength of solution is generally more limiting than energy in these environments, as there can be sulfur, methane, hydrogen and CO_2_ present, depending on host lithology.

### Titan

Titan is a cold, hydrocarbon-rich extraterrestrial body, so cold subsurface environments rich in hydrocarbons are possible analogs for this target. Titan has an atmosphere assumed to contain primarily N_2_, CH_4_ and H_2_, surface temperatures of ∼90 K and a surface pressure of ∼1.46 bar (Jakosky et al., 2003; Niemann et al., 2005; Jennings et al., 2009). It has liquid reservoirs of N_2_, CH_4_, Ar, CO, C_2_H_2_–C_4_H_10_, and H_2_ (Cordier et al., 2012, 2013) in addition to other complex C–H and C–N chains (Desai et al., 2017) on the surface, which possibly permeate the subsurface (Hayes et al., 2008; Mousis et al., 2014). The temperature of Titan’s surface precludes all but the most psychrophilic lifestyles, though there are indications that life at these temperatures is within the realms of possibility (Price and Sowers, 2004; Panikov and Sizova, 2007; Amato and Christner, 2009). Evidence of cryovolcanism also indicates possible areas of warmer temperatures (Lopes et al., 2013). Conditions of Titan are likely to increase reactivity of silicon compounds, leading to some speculation on the possibility of silicon-based life in such conditions (Bains, 2004).

Subsurface hydrocarbon deposits are proposed as possible analogs for Titan (L’Haridon et al., 1995). Microbial life in these environments metabolize hydrocarbons under challenging conditions of temperature, pressure and oxygen limitation, in addition to limitations imposed by the properties of hydrocarbons as substrates. However, there is little information on minimum temperatures in hydrocarbon deposits on earth. Water is important in these hydrocarbon reservoirs, with a positive correlation between cell activity and water availability (Head et al., 2003; Larter et al., 2006); however temperatures on Titan likely limit the availability of this solvent to warmer areas linked to cryovolcanism (Kargel, 1994; Lopes et al., 2013). Methane has been proposed as a possible alternative solvent for Titan (Stofan et al., 2007), though its chemical properties are somewhat different from water. The conditions on Titan as currently understood allow for more extensive gas hydrate formation, including in the subsurface and on the surface (Osegovic and Max, 2005; Fortes et al., 2007). Methane hydrates and associated microbial communities form on Earth in deep sediment and permafrost (Osegovic and Max, 2005).

## Energetics and the Subsurface

### Growth, Activity, and Dormancy

The physiological state of microorganisms in the subsurface can be grouped into three categories based on metabolic activity: (1) *Growth*, where the energy/nutrient demands of the cell are met, and there is sufficient energy for cell division and biomolecule synthesis; (2) *basal maintenance* (alternatively termed vegetative) in which cell division is not occurring, but cells are carrying out essential housekeeping functions for cell viability such as repair and replacement of biomolecules, and maintenance of membrane integrity (Hoehler and Jørgensen, 2013); and (3) *dormancy* (endo-spores), a reversible state of low to zero metabolic activity that is generally thought to be an evolutionary strategy to overcome unfavorable conditions for growth (Jones and Lennon, 2010; Lennon and Jones, 2011; Bradley et al., 2018). The majority of microorganisms in most environmental systems spend their time in non-dividing and energy limited states (Bergkessel et al., 2016). Environmental and energetic cues causing microorganisms to switch between growth, activity and dormancy are unclear, though these physiological states have important implications for the evolution and ecology of low energy subsurface settings.

Many microorganisms on Earth are capable of temporarily resisting stresses such as temperature, desiccation and antibiotics by entering resting states or by forming spores (Jones and Lennon, 2010). These dormant microorganisms act as a seed bank, contributing to future microbial diversity when conditions become favorable again. Dormancy might be a relevant life strategy for considering life on planetary bodies with possible past habitable conditions or where environmental conditions fluctuate temporally. Due to the estimated life-span of viability for an endospore and measured endospore abundance with depth in subseafloor sediments, one strategy for extended longevity in the subsurface appears to be periodic germination of spores to carry out repair functions (Braun et al., 2017). Nonetheless, long term survival on geological timescales through low metabolic activity may be superior to dormancy since a minimum metabolic activity for maintenance is required to counteract damage to biomolecules accumulated over time caused by background radiation, hydrolysis, oxidation, etc. (Johnson et al., 2007).

Energy requirements of growth, maintenance activities and dormancy are difficult to directly measure, but is estimated to differ by orders of magnitude (10^6^: 10^3^: 1) (Price and Sowers, 2004). Investigations into limits of energy required for growth in cultured microbes vary from −20 to −9 kJ mol^−1^ of energy as a limiting threshold for actively growing populations (Schink, 1997; Hoehler, 2004; Schink and Stams, 2006). Values for *in situ* investigations are lower again, with values of 190 zeptoWatts (zW) per cell in ultra-oligotrophic sediments and theoretical values as low as 1 zW per cell (LaRowe and Amend, 2015a). For comparison, a J mol^−1^ is a unit of energy whereas W is a unit of power, which is energy use over time. Therefore, 190 zW is equivalent to 1.9 × 10^−22^ kJ mol^−1^ s^−1^. More recent work suggests that −20 to −10 kJ mol^−1^ is the minimum energy required for ATP synthesis (Müller and Hess, 2017), suggesting that lower values are more representative of cells under vegetative states. However, modeling energy requirements of *in situ* populations of cells requires several assumptions about growth requirements such as cellular biomolecule content, cell size and microbial taxa (Lever et al., 2015; Bergkessel et al., 2016; Kempes et al., 2017). Likewise, the molecular mechanisms and physiological characteristics associated with non-growth activity in cultured microorganisms remains poorly understood, due partly to the challenges associated with controlling, reproducing, and measuring non-growing states (Bergkessel et al., 2016). It is unclear how mechanisms (such as those reviewed in Lever et al., 2015) and energy consumption in generally fast-growing, mesophilic model microorganisms relate to those employed by extremophiles such as those of low energy subsurface environments.

Despite low energy and nutrients, multiple lines of evidence indicate active microbial life in the subsurface. These include rates of metabolic sulfate reduction and methane cycling inferred from geochemical profiles in environmental samples, and activity measurements in microcosm experiments through radioisotope and stable isotope labeling of compounds (Morono et al., 2011; Wegener et al., 2012; Orcutt et al., 2013a; Glombitza et al., 2016; Robador et al., 2016b; Trembath-Reichert et al., 2017). Detection of bulk transcriptional activity and translational activity has the advantage of ascribing taxonomy and function to active microbiota (Orsi et al., 2013, 2016; Hatzenpichler et al., 2016; Marlow et al., 2016). Calorimetry measurements detected microbial activity in oceanic crustal fluids, measuring cellular energy consumption ranging from 0.2 to 5.7 pW cell^−1^ (Robador et al., 2016a). Additionally, highly sensitive techniques such as nanometer-scale secondary ion mass spectrometry (nanoSIMS), and bioorthogonal non-canonical amino acid tagging (BONCAT), have detected activity down to the single cell level in slow growing microorganisms (Morono et al., 2011; Hatzenpichler et al., 2016; Trembath-Reichert et al., 2017), providing insight into individual cell-to-cell variation in metabolism in low energy settings. Such activity measurements have led to proposed cell turnover rates of months to 10s of 1000s of years (Phelps et al., 1994; Biddle et al., 2006; Hoehler and Jørgensen, 2013; Braun et al., 2017; Trembath-Reichert et al., 2017).

### Energy Yield of Various Redox Reactions in the Low Energy Subsurface and on Extraterrestrial Environments

According to the “follow the energy” approach to identifying habitable zones (Hoehler, 2007), electron acceptors and donors must be present in large enough quantities, and the energy released needs to be sufficient for life to make use of it (Nixon et al., 2012). The energy of a reaction differs according to environmental conditions, particularly in “extreme” environments such as the subsurface and current potential extraterrestrial targets, where temperature, pressure, pH and concentration of available reactants and products deviate significantly from standard conditions of 25°C, 1 atm, 1 M substrate concentration (D’Hondt, 2002; Hoehler, 2004, 2007; LaRowe and Van Cappellen, 2011; Bradley et al., 2018). Additionally, Gibbs free energy yield calculations for various reactions give only the maximum theoretical available energy at a given set of conditions. Calculations of the energy yield of a reaction (i.e., kJ per mole of substrate) should be considered against the availability of the substrate to consider energy yield in a given volume of the environment (i.e., kJ per liter) (LaRowe and Van Cappellen, 2011; LaRowe and Amend, 2015b; Orcutt et al., 2015). This volumetric energy yield is particularly relevant in subsurface sites, where certain electron acceptors and donors can be in short supply.

To evaluate the feasibility of various redox reactions in low energy subsurface analog sites and extraterrestrial targets (**Table 1**), we calculated the Gibbs free energy yield of reactions under *in situ* conditions for a suite of reactions (**Table 2**), following approaches outlined in detail elsewhere (Amend and Shock, 2001; Osburn et al., 2014). We scaled the activity coefficients in the Gibbs free energy equations to account for ionic strength in the various environments, as well as temperature effects on standard conditions; pressure effects were not included as these have far less of an impact than temperature (Amend and Shock, 2001). Where available, we used measured or interpreted *in situ* concentrations of reactants and products available from the primary literature (**Supplementary Table S1**). Care was taken to select subsurface values wherever possible, particularly for sites such as Rio Tinto that have surface components. Where concentrations were unknown, we assumed an end-member limiting concentration of 1 nmol substrate per liter (**Supplementary Table S1**). Gibbs free energy yields were normalized to the number of electrons exchanged in the reaction, to calculate energy yields as kJ per mole of electrons for cross comparison of reactions. The energy density of the selected reactions in an environment were calculated by scaling the reactions to a mole of the limiting reactant, which was assumed to be the electron donor in all cases. While this assumption is not always true, such as in organic rich marine sediment where electron acceptors become limiting (Orcutt et al., 2011), its use allows more direct comparison within the presented dataset and other work (Osburn et al., 2014). Finally, we summed the free energy available from all calculated reactions together to compare sites to one another, although this presents an overestimate as there would be competing reactions for some substrates.

**Table 1:** Extraterrestrial and Earth low energy subsurface analog sites considered in energy calculations.

Site	Overview of site characteristics
**Extraterrestrial**	
Mars	Low estimate
	High estimate
Enceladus	Hypothesized crustal seafloor-liquid interface
Europa	Hypothesized crustal seafloor-liquid interface
Titan	Surface
**Marine**	
North Pond	Basaltic crust, cool, and oxic
Juan de Fuca	Basaltic crust, warm, and anoxic
Lost City	Ultramafic crust, warm hydrothermal vents
South Pacific Gyre	Extremely oligotrophic, oxic sediment
Gulf of Mexico	Cold anoxic brine seeps
**Continental**	
Sanford Underground Research Facility	Metamorphic crust
Mont Terri	Opalinus clay
Rio Tinto	Massive pyrite ore deposit
University Valley	Polar desert permafrost, low estimate
	Polar desert permafrost, high estimate
Atacama	Hyperarid desert, low temperature, high pH
	Hyperarid desert, high temperature, low pH
Lake Vida	Ice-enclosed hypersaline lake

*Environmental concentrations (listed in **Supplementary Table S1**) from references listed in **Supplementary Table S2**.*

**Table 2:** Reactions considered in Gibbs free energy and energy density calculations.

Redox pair	Equation
H_2_/O_2_	H_2(aq)_ + 0.5O_2(aq)_ → H_2_O_(l)_
H_2_/${\text{NO}}_{3}^{-}$ (${\text{NO}}_{2}^{-}$)	H_2(aq)_ + ${\text{NO}}_{3}^{-}$ → ${\text{NO}}_{2}^{-}$ + H_2_O_(l)_
H_2_/${\text{NO}}_{3}^{-}$ (NH_3_)	4H_2(aq)_ + ${\text{NO}}_{3}^{-}$ + H^+^ → NH_3(aq)_ + 3H_2_O_(l)_
H_2_/${\text{SO}}_{4}^{2}$	4H_2(aq)_ + ${\text{SO}}_{4}^{2-}$ + 2H^+^ → H_2_S_(aq)_ + 4H_2_O_(l)_
H_2_/CO_2_	4H_2(aq)_ + CO_2__(aq)_ → CH_4(aq)_ + 2H_2_O_(l)_
H_2_S/O_2_	H_2_S_(aq)_ + 2O_2(aq)_ → ${\text{SO}}_{4}^{2-}$ + 2H^+^
H_2_S/${\text{NO}}_{3}^{-}$	5H_2_S_(aq)_ + 8${\text{NO}}_{3}^{-}$ → 4N_2(aq)_ + 5${\text{SO}}_{4}^{2-}$ + 4H_2_O_(l)_ + 2H^+^
Fe^2+^/O_2_	2Fe^2+^ + 0.5O_2(aq)_ + 2H^+^ → 2Fe^3+^+ H_2_O_(l)_
FeS_2_/O_2_	FeS_2(s)_ + 3.5O_2(aq)_ + H_2_O_(l)_ → Fe^2+^ + 2${\text{SO}}_{4}^{2-}$ + 2H^+^
NH_3_/O_2_	NH_3(aq)_ + 1.5O_2(aq)_ → ${\text{NO}}_{2}^{-}$ + H_2_O_(l)_ + H^+^
NH_3_/${\text{NO}}_{2}^{-}$	NH_3(aq)_ + ${\text{NO}}_{2}^{-}$ + H^+^→ N_2(aq)_ + 2H_2_O_(l)_
NH_3_/${\text{SO}}_{4}^{2-}$	NH_3(aq)_ + ${\text{SO}}_{4}^{2-}$ + H^+^ → ${\text{NO}}_{3}^{-}$ + H_2_S_(aq)_ + H_2_O_(l)_
CH_4_/O_2_	CH_4(aq)_ + 2O_2(aq)_ → CO_2(aq)_ + 2H_2_O_(l)_
CH_4_/${\text{NO}}_{3}^{-}$	CH_4(aq)_ + 4${\text{NO}}_{3}^{-}$ → 4${\text{NO}}_{2}^{-}$ + CO_2(aq)_ + H_2_O_(l)_
CH_4_/${\text{SO}}_{4}^{2-}$	CH_4(aq)_ + ${\text{SO}}_{4}^{2-}$ + 2H^+^ → H_2_S_(aq)_ + CO_2(aq)_ + 2H_2_O_(l)_

*Note that redox pairs of Fe^2+^ with ${\text{NO}}_{3}^{-}$ or ${\text{SO}}_{4}^{2-}$ were also considered, but as the reactions were always endergonic (see **Supplementary Table S1**), they were excluded from further presentation.*

**FIGURE 3:**
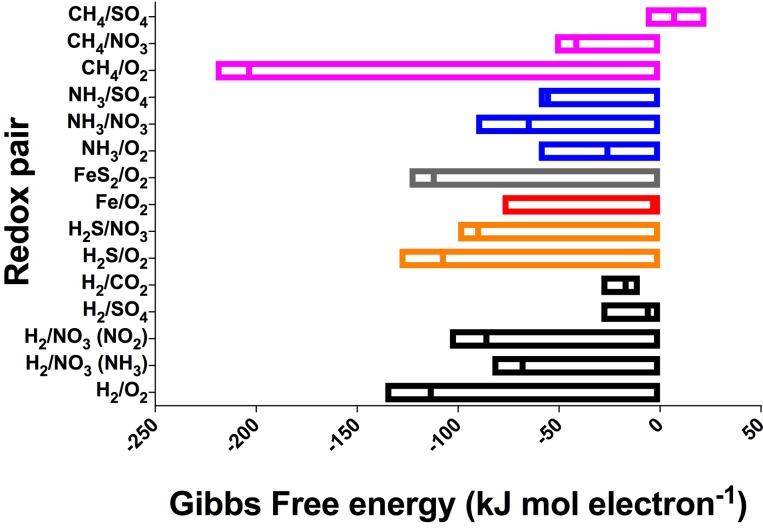
Range and mean of Gibbs Free energy yields, normalized to kJ per mole electron transferred per reaction, across all sites for the redox pairs listed in **Table 1**. Negative values indicate exergonic reactions. See **Supplementary Table S1** for all values. Reactions grouped by electron donor and color (pink, CH_4_; blue, NH_3_; gray, FeS_2_; red, Fe^2+^; orange, H_2_S; black, H_2_).

Despite the large variation in environmental conditions – such as temperature, ionic strength, and concentrations – our calculations show that there is remarkable consistency in the energy yields per electron transferred across all subsurface and extraterrestrial sites considered (**Table 1**), with most reactions varying by less than 100 kJ mol electron^−1^ (**Figure 3** and **Supplementary Table S1**). The most variable reaction energetic yield is the CH_4_/O_2_ redox pair, whereas the least variable is the H_2_/CO_2_ pair. Only the CH_4_/SO_4_ redox pair is expected to be endergonic under some conditions (though just barely exergonic and at or below the theoretical minimum energy limit in others); all other reactions are estimated to be exergonic under all conditions considered (note that calculations for Fe^2+^ paired with either ${\text{NO}}_{3}^{-}$ or ${\text{SO}}_{4}^{2-}$ was endergonic under all conditions and was excluded from further analysis). This indicates that a wide range of redox reactions could theoretically be supported on the extraterrestrial targets, and that the energy yields can be similar to what can be found in Earth’s subsurface environments. However, given that the presence of some electron donors and acceptors in extraterrestrial targets are poorly constrained [e.g., unknown electron acceptors on Enceladus, despite confirmation of electron donors methane, ammonium, and possibly hydrogen (Postberg et al., 2009; Waite et al., 2009)], theoretical feasibility needs to be constrained by probability of both electron donors and acceptors being present.

**FIGURE 4:**
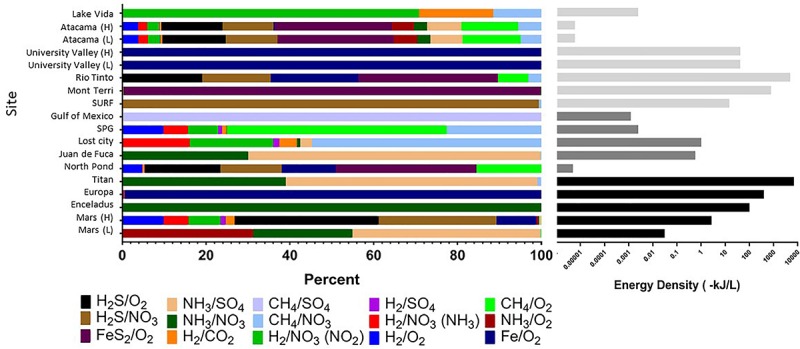
Cumulative volumetric energy densities of redox reactions per site based on environmental concentrations of variables in each reaction (**Table 1**). **(Left)** Shows the percent contribution of various reactions as shown in the color legend. **(Right)** Shows combined absolute energy density (as kJ per liter) for all reactions, with gray scale reflecting habitat type. All calculations assumed that the electron donor was the limiting substrate. See **Supplementary Table S1** for all values and formulas.

Strikingly, extraterrestrial sites are predicted to have similar cumulative energy densities as Earth’s subsurface habitats (with conservative assumptions about electron donor and acceptor concentrations), although the dominant energy-rich processes vary (**Figure 4** and Supplementary Table S1). For example, cumulative volumetric energy densities on Mars are estimated to range from 0.03 to 3 kJ L^-1^, supported primarily by the electron donors NH_3_, H_2_S, or hydrogen reacting with sulfate, nitrate, or oxygen, depending on the scenario chosen for electron donor concentration, pH, and temperature. Under the scenario of low electron donor concentration, low pH, and low temperature, the predicted Martian energy density and dominant reactions are similar to those observed at the Earth analog site at the Juan de Fuca Ridge flank subsurface oceanic crust. Under the scenario of higher electron donor concentrations, pH, and temperature, the cumulative volumetric energy density and dominant reactions estimate is more similar to what is estimated from the Earth analog sites in the Rio Tinto. The base of the presumed Europan ocean has an estimated energy density of 400 kJ L^-1^ fueled primarily by iron oxidation, if dissolved oxygen is present (Teolis et al., 2017) and penetrates to the water-rock interface and if iron is released from water-rock reactions. This volumetric energy density and dominant reaction pattern is similar to that estimated for the Earth analog site at University Valley. By contrast, the ocean on Enceladus is estimated to have an energy density of 100 kJ L^-1^ fueled by ammonia oxidation with nitrate; none of our comparison Earth analog sites had similar energy density estimates from this reaction. The cumulative volumetric energy density estimates for Titan are the highest we estimate in this exercise, fueled by ammonia oxidation with sulfate or nitrate in a similar pattern as estimated for the Juan de Fuca analog system, but we highlight that this is the least well constrained system. Overall, although based on poorly constrained concentrations, these projections indicate that extraterrestrial sites could have sufficient overall energy to host chemolithotrophic communities.

The predicted relative contribution of each redox pair to each site is applicable information for the “follow the energy” approach to habitability (Hoehler, 2007), and can further be constrained by comparison studies of microbial metabolic processes in the Earth analog systems, to see if the predicted energy rich metabolisms are indeed those that occur. This approach of comparing energy density to microbial community function has recently been shown for some subsurface sites (Osburn et al., 2014; Reveillaud et al., 2016; Momper et al., 2017), demonstrating the power of this energy density approach to be a useful predictor of metabolic function. For example, North Pond energy is primarily from the FeS_2_/O_2_ couple (**Figure 4**), indicating that solid mineral substrates may be significant in this environment. Oxidation of hydrogen sulfide is also predicted to yield more energy than other electron donors (**Figure 4**), which agrees well with information on metabolic function in the community indicating that sulfur oxidizers are present in greater relative abundance as compared to hydrogen, ammonia and nitrite metabolisms (Jørgensen and Zhao, 2016; Meyer et al., 2016). Lost City estimates showmethane and hydrogen oxidation reactions as significant sources of energy (**Figure 4**), which agrees with work indicating methane oxidizers are common in this system but contrasts with other recent work pointing to sulfate metabolisms as being more important than hydrogen metabolisms in this environment (Lang et al., 2018). At this site, the Gibbs free energy of the H_2_/CO_2_ couple is relatively high but the energy density low (**Figures 3, 4**), as dissolved CO_2_ concentration is scarce because it rapidly precipitates as carbonates in the high pH environment. As shown previously, sulfide oxidizing metabolisms are energy rich in the continental subsurface at the Sanford Underground Research Facility, and sulfide oxidizers are dominant in the microbial community (Osburn et al., 2014; Momper et al., 2017). In the subsurface portion of Rio Tinto, observation of iron and sulfur metabolisms matches with estimates of energy density (García-Moyano et al., 2012; Sánchez-Andrea et al., 2012; Amils et al., 2014). The Atacama analog site has a very low predicted energy availability, although we note that factors like water availability may be more important than energy availability in structuring the microbial community at the hyperarid and polar desert environments (Goordial et al., 2016). It is notable that the range of pH and temperature scenarios at the Atacama and University Valley sites did not particularly affect the predicted dominant reactions or volumetric energy densities at the hyper-arid sites, unlike the Mars sites, which notably changed, highlighting that the ion concentrations are key for determining dominant reactions and energy densities. Overall, this “follow the energy” approach of matching predicting energy density to microbial community structure and function may inform the likely metabolisms that might be found on extraterrestrial targets.

As with all such calculations however, there are caveats to the estimates presented. Many of these values are estimates, due to missing information for many of the extraterrestrial targets (**Supplementary Table S1**). The calculations assume steady state concentrations, whereas in Earth analog environments, current concentrations do not reflect the energy available from biogeochemical reactions that may have already occurred. Likewise, these steady state calculations do not consider the fluxes of electron donors or acceptors, which will also have strong influence on energetics. The calculations assume a cumulative energy from many different reactions that would consume the same electron donors and acceptors and do not take into account competition for these substrates between the reactions, nor the possible variabilities in reaction kinetics, which is beyond the scope of this paper. The extraterrestrial calculations were generalized across the entire target and not modified for variations in possible habitat type. These calculations also do not take any other habitability factor (i.e., **Figure 1**) such as water, carbon availability and toxicity into consideration, though these also affect microbial communities in subsurface analog sites (Goordial et al., 2016; Purkamo et al., 2017). These speculations assume that limits of energy of life in earth systems would be applicable elsewhere. This is a rather significant assumption, but a key part of extraterrestrial-analog investigation in general. Finally, it must be noted that habitability does not necessarily mean a set of conditions will be inhabited (Cockell et al., 2016). With these limitations in mind, these figures nevertheless serve to refine the question of habitability in combination with other factors (**Figure 1**) by giving a rough indication of which microbial metabolic redox reactions in various deep subsurface sites and potential extraterrestrial targets potentially provide sufficient energy for life.

In conclusion, the low energy subsurface is a collective term for environments that are relatively isolated from surface processes, though the exact habitable range is yet largely unconstrained. The largely prokaryotic life in these environments survive a range of “extreme” conditions such as temperature and availability of electron donors or acceptors. These factors have a direct effect on the available energy of a redox reaction, which in turn affects the viability of a particular metabolism in a given set of environmental conditions. These characteristics make the low energy subsurface a source of potential analogs in the search for life elsewhere, as this combination of conditions and resource scarcity are useful in the search for the boundaries of where life is possible across a broad spectrum of possible extreme environments. Therefore, studying the subsurface in the context of analogs contributes to constraining the energetic boundaries of “following the energy” as an approach to the search for life.

## Author Contributions

RJ and BO developed the idea and calculated values. RJ and JG reviewed the literature. RJ wrote the paper with input from all coauthors.

## Conflict of Interest Statement

The authors declare that the research was conducted in the absence of any commercial or financial relationships that could be construed as a potential conflict of interest.

## Supplementary Material

TABLE S1 Environmental values and equations used to calculate Gibbs free energy and energy density calculations.

TABLE S2 Source references for values in **Supplementary Table S1**.

## References

[B1] Amato P. Christner B. C. (2009). Energy metabolism response to low-temperature and frozen conditions in *Psychrobacter cryohalolentis*. *Appl. Environ. Microbiol.* 75 711–718. 10.1128/AEM.02193-08 19060163 PMC2632129

[B2] Amenabar M. J. Shock E. L. Roden E. E. Peters J. W. Boyd E. S. (2017). Microbial substrate preference dictated by energy demand rather than supply. *Nat. Geosci.* 10 577–581. 10.1038/ngeo2978 30944580 PMC6443248

[B4] Amend J. P. Shock E. L. (2001). Energetics of overall metabolic reactions of thermophilic and hyperthermophilic Archaea and Bacteria. *FEMS Microbiol. Rev.* 25 175–243. 11250035 10.1111/j.1574-6976.2001.tb00576.x

[B5] Amend J. P. Teske A. (2005). Expanding frontiers in deep subsurface microbiology. *Palaeogeogr. Palaeoclimatol. Palaeoecol.* 219 131–155. 10.1016/j.palaeo.2004.10.018

[B6] Amils R. Fernández-Remolar D. C. IPBSL Team (2014). Río Tinto: a geochemical and mineralogical terrestrial analogue of mars. *Life* 4 511–534. 10.3390/life4030511 25370383 PMC4206857

[B7] Anraku Y. (1988). Bacterial electron transport Chains. *Annu. Rev. Biochem.* 57 101–132. 10.1146/annurev.biochem.57.1.101 3052268

[B8] Antunes A. Ngugi D. K. Stingl U. (2011). Microbiology of the Red Sea (and other) deep-sea anoxic brine lakes. *Environ. Microbiol. Rep.* 3 416–433. 10.1111/j.1758-2229.2011.00264.x 23761304

[B9] Arndt S. Barker Jørgensen B. LaRowe D. E. Middelburg J. J. Pancost R. D. Regnier P. (2013). Quantifying the degradation of organic matter in marine sediments: a review and synthesis. *Earth Sci. Rev.* 123 53–86. 10.1016/j.earscirev.2013.02.008

[B10] Bach W. (2016). Some compositional and kinetic controls on the bioenergetic landscapes in oceanic basement. *Front. Microbiol.* 7:107. 10.3389/fmicb.2016.00107 26903986 PMC4746428

[B11] Bach W. Edwards K. J. (2003). Iron and sulfide oxidation within the basaltic ocean crust: Implications for chemolithoautotrophic microbial biomass production. *Geochim. Cosmochim. Acta* 67 3871–3887. 10.1016/S0016-7037(03)00304-1

[B12] Badalamenti J. P. Summers Z. M. Chan C. H. Gralnick J. A. Bond D. R. (2016). Isolation and genomic characterization of “*Desulfuromonas soudanensis* WTL”, a metal- and electrode-respiring bacterium from anoxic deep subsurface brine. *Front. Microbiol.* 7:913 10.3389/fmicb.2016.00913 PMC4914508 27445996

[B13] Bagnoud A. de Bruijn I. Andersson A. F. Diomidis N. Leupin O. X. Schwyn B. (2016). A minimalistic microbial food web in an excavated deep subsurface clay rock. *FEMS Microbiol. Ecol.* 92:fiv138. 10.1093/femsec/fiv138 26542073

[B14] Bains W. (2004). Many chemistries could be used to build living systems. *Astrobiology* 4 137–167. 10.1089/153110704323175124 15253836

[B15] Baker B. J. Saw J. H. Lind A. E. Lazar C. S. Hinrichs K.-U. Teske A. P. (2016). Genomic inference of the metabolism of cosmopolitan subsurface Archaea, Hadesarchaea. *Nat. Microbiol.* 1:16002. 10.1038/nmicrobiol.2016.2 27572167

[B16] Bakermans C. Skidmore M. L. Douglas S. McKay C. P. (2014). Molecular characterization of bacteria from permafrost of the Taylor Valley, Antarctica. *FEMS Microbiol. Ecol.* 89 331–346. 10.1111/1574-6941.12310 24592998

[B17] Barge L. M. White L. M. (2017). Experimentally testing hydrothermal vent origin of life on enceladus and other Icy/ocean worlds. *Astrobiology* 47 820–833. 10.1089/ast.2016.1633 28836818

[B18] Bartlett D. H. Bidle K. A. (1999). *Enigmatic Microorganism and Life in Extreme Environments.* Dordrecht: Kluwer Academic, 502–509. 10.1017/CBO9781107415324.004

[B20] Beatty J. T. Overmann J. Lince M. T. Manske A. K. Lang A. S. Blankenship R. E. (2005). An obligately photosynthetic bacterial anaerobe from a deep-sea hydrothermal vent. *Proc. Natl. Acad. Sci. U.S.A.* 102 9306–9310. 10.1073/pnas.0503674102 15967984 PMC1166624

[B21] Becker K. Fisher A. T. (2000). Permeability of upper oceanic basement on the eastern flank of the Juan de Fuca Ridge determined with drill-string packer experiments. *J. Geophys. Res. Atmos.* 105 897–912. 10.1029/1999jb900250

[B22] Bell R. E. Studinger M. Tikku A. A. Clarke G. K. C. Gutner M. M. Meertens C. (2002). Origin and fate of Lake Vostok water frozen to the base of the East Antarctic ice sheet. *Nature* 416 307–310. 10.1038/416307a 11907573

[B23] Beller H. R. Zhou P. Legler T. C. Chakicherla A. Kane S. Letain T. E. (2013). Genome-enabled studies of anaerobic, nitrate-dependent oxidation in the chemolithoautotrophic bacterium *Thiobacillus denitrificans*. *Front. Microbiol.* 4:249. 10.3389/fmicb.2013.00249 24065960 PMC3753534

[B24] Bergkessel M. Basta D. W. Newman D. K. (2016). The physiology of growth arrest: uniting molecular and environmental microbiology. *Nat. Rev. Microbiol.* 14 549–562. 10.1038/nrmicro.2016.107 27510862 PMC10069271

[B25] Biddle J. F. Lipp J. S. Lever M. A. Lloyd K. G. Sorensen K. B. Anderson R. (2006). Heterotrophic Archaea dominate sedimentary subsurface ecosystems off Peru. *Proc. Natl. Acad. Sci. U.S.A.* 103 3846–3851. 10.1073/pnas.0600035103 16505362 PMC1533785

[B26] Bradley J. A. Amend J. P. LaRowe D. E. (2018). Bioenergetic controls on microbial ecophysiology in marine sediments. *Front. Microbiol.* 9:180. 10.3389/fmicb.2018.00180 29487581 PMC5816797

[B27] Braun S. Mhatre S. S. Jaussi M. Røy H. Kjeldsen K. U. Pearce C. (2017). Microbial turnover times in the deep seabed studied by amino acid racemization modelling. *Sci. Rep.* 7:5680. 10.1038/s41598-017-05972-z 28720809 PMC5516024

[B28] Brazelton W. J. Morrill P. L. Szponar N. Schrenk M. O. (2013). Bacterial communities associated with subsurface geochemical processes in continental serpentinite springs. *Appl. Environ. Microbiol.* 79 3906–3916. 10.1128/AEM.00330-13 23584766 PMC3697581

[B29] Brazelton W. J. Nelson B. Schrenk M. O. (2012). Metagenomic evidence for H_2_ oxidation and H_2_ production by serpentinite-hosted subsurface microbial communities. *Front. Microbiol.* 2:268 10.3389/fmicb.2011.00268 PMC3252642 22232619

[B30] Brazelton W. J. Schrenk M. O. Kelley D. S. Baross J. A. (2006). Methane- and sulfur-metabolizing microbial communities dominate the lost city hydrothermal field ecosystem. *Appl. Environ. Microbiol.* 72 6257–6270. 10.1128/AEM.00574-06 16957253 PMC1563643

[B31] Bulat S. A. (2016). Microbiology of the subglacial Lake Vostok: first results of borehole-frozen lake water analysis and prospects for searching for lake inhabitants. *Philos. Trans. R. Soc. A Math. Phys. Eng. Sci.* 374:20140292. 10.1098/rsta.2014.0292 26667905

[B32] Byrne S. Dundas C. M. Kennedy M. R. Mellon M. T. McEwen A. S. Cull S. C. (2009). Distribution of mid-latitude ground ice on Mars from new impact craters. *Science* 325 1674–1676. 10.1126/science.1175307 19779195

[B33] Carere C. R. Hards K. Houghton K. M. Power J. F. McDonald B. Collet C. (2017). Mixotrophy drives niche expansion of verrucomicrobial methanotrophs. *ISME J.* 11 2599–2610. 10.1038/ismej.2017.112 28777381 PMC5649168

[B34] Carlson R. W. Johnson R. E. Anderson M. S. (1999). Sulfuric acid on europa and the radiolytic sulfur cycle. *Science* 286 97–99. 10.1126/science.286.5437.97 10506568

[B35] Catling D. C. Claire M. W. Zahnle K. J. Quinn R. C. Clark B. C. Hecht M. H. (2010). Atmospheric origins of perchlorate on Mars and in the Atacama. *J. Geophys. Res.* 115:E00E11 10.1029/2009JE003425

[B36] Chapelle F. H. O’Neill K. Bradley P. M. Methé B. A. Ciufo S. A. Knobel L. L. (2002). A hydrogen-based subsurface microbial community dominated by methanogens. *Nature* 415 312–315. 10.1038/415312a 11797006

[B37] Chen L.-X. Huang L.-N. Méndez-García C. Kuang J.-L. Hua Z.-S. Liu J. (2016). Microbial communities, processes and functions in acid mine drainage ecosystems. *Curr. Opin. Biotechnol.* 38 150–158. 10.1016/j.copbio.2016.01.013 26921733

[B38] Clennell M. B. Hovland M. Booth J. S. Henry P. Winters W. J. (1999). Formation of natural gas hydrates in marine sediments: 1. Conceptual model of gas hydrate growth conditioned by host sediment properties. *J. Geophys. Res. Solid Earth* 104 22985–23003. 10.1029/1999JB900175

[B39] Clifford S. M. Lasue J. Heggy E. Boisson J. McGovern P. Max M. D. (2010). Depth of the martian cryosphere: revised estimates and implications for the existence and detection of subpermafrost groundwater. *J. Geophys. Res. Planets* 115 10.1029/2009JE003462

[B40] Clifford S. M. Parker T. J. (2001). The evolution of the martian hydrosphere: implications for the fate of a primordial ocean and the current state of the northern plains. *Icarus* 154 40–79. 10.1006/icar.2001.6671

[B41] Cockell C. S. Bush T. Bryce C. Direito S. Fox-Powell M. G. Harrison J. P. (2016). Habitability: a review. *Astrobiology* 16 89–117. 10.1089/ast.2015.1295 26741054

[B42] Cogley J. G. (1984). Continental margins and the extent and number of the continents. *Rev. Geophys.* 22 101–122. 10.1029/RG022i002p00101

[B43] Colman D. R. Poudel S. Stamps B. W. Boyd E. S. Spear J. R. (2017). The deep, hot biosphere: twenty-five years of retrospection. *Proc. Natl. Acad. Sci. U.S.A.* 114 6895–6903. 10.1073/pnas.1701266114 28674200 PMC5502609

[B44] Cordier D. Mousis O. Lunine J. I. Lavvas P. Vuitton V. (2013). Erratum: an estimate of the chemical composition of titan’s lakes. *Astrophys. J. Lett.* 768 L23–L26. 10.1088/2041-8205/768/1/L23

[B45] Cordier D. Mousis O. Lunine J. I. Lebonnois S. Rannou P. Lavvas P. (2012). Titan’s lakes chemical composition: Sources of uncertainties and variability. *Planet. Space Sci.* 61 99–107. 10.1016/j.pss.2011.05.009

[B46] Crits-Christoph A. Robinson C. K. Barnum T. P. Fricke W. Davila A. F. Jedynak B. (2013). Colonization patterns of soil microbial communities in the Atacama Desert. *Microbiome* 1:28. 10.1186/2049-2618-1-28 24451153 PMC3971613

[B47] Daly R. A. Borton M. A. Wilkins M. J. Hoyt D. W. Kountz D. J. Wolfe R. A. (2016). Microbial metabolisms in a 2.5-km-deep ecosystem created by hydraulic fracturing in shales. *Nat. Microbiol.* 1:16146. 10.1038/nmicrobiol.2016.146 27595198

[B48] Desai R. T. Coates A. J. Wellbrock A. Vuitton V. Crary F. J. González-Caniulef D. (2017). Carbon chain anions and the growth of complex organic molecules in Titan’s ionosphere. *Astrophys. J.* 844:L18 10.3847/2041-8213/aa7851

[B49] D’Hondt S. (2002). Metabolic activity of subsurface life in deep-sea sediments. *Science* 295 2067–2070. 10.1126/science.1064878 11896277

[B50] D’Hondt S. Inagaki F. Zarikian C. A. Abrams L. J. Dubois N. Engelhardt T. (2015). Presence of oxygen and aerobic communities from sea floor to basement in deep-sea sediments. *Nat. Geosci.* 8 299–304. 10.1038/ngeo2387

[B51] D’Hondt S. Spivack A. J. Pockalny R. Ferdelman T. G. Fischer J. P. Kallmeyer J. (2009). Subseafloor sedimentary life in the South Pacific Gyre. *Proc. Natl. Acad. Sci. U.S.A.* 106 11651–11656. 10.1073/pnas.0811793106 19561304 PMC2702254

[B52] Dick G. J. Anantharaman K. Baker B. J. Li M. Reed D. C. Sheik C. S. (2013). The microbiology of deep-sea hydrothermal vent plumes: Ecological and biogeographic linkages to seafloor and water column habitats. *Front. Microbiol.* 4:124. 10.3389/fmicb.2013.00124 23720658 PMC3659317

[B53] Dilek Y. Furnes H. (2014). Ophiolites and their origins. *Elements* 10 93–100. 10.2113/gselements.10.2.93

[B54] Domagal-Goldman S. D. Wright K. E. Adamala K. Arina de la Rubia L. Bond J. Dartnell L. R. (2016). The astrobiology primer v2.0. *Astrobiology* 16 561–653. 10.1089/ast.2015.1460 27532777 PMC5008114

[B55] Dopson M. Johnson D. B. (2012). Biodiversity, metabolism and applications of acidophilic sulfur-metabolizing microorganisms. *Environ. Microbiol.* 14 2620–2631. 10.1111/j.1462-2920.2012.02749.x 22510111

[B56] Druschel G. K. Baker B. J. Gihring T. M. Banfield J. F. (2004). Acid mine drainage biogeochemistry at Iron Mountain, California. *Geochem. Trans.* 5 13–32. 10.1063/1.1769131 PMC1475782 35412773

[B57] Dzaugis M. E. Spivack A. J. Dunlea A. G. Murray R. W. D’Hondt S. (2016). Radiolytic hydrogen production in the subseafloor basaltic aquifer. *Front. Microbiol.* 7:76. 10.3389/fmicb.2016.00076 26870029 PMC4740390

[B58] Edwards K. J. Bach W. McCollom T. M. (2005). Geomicrobiology in oceanography: microbe-mineral interactions at and below the seafloor. *Trends Microbiol.* 13 449–456. 10.1016/j.tim.2005.07.005 16054363

[B59] Edwards K. J. Becker K. Colwell F. (2012a). The deep, dark energy biosphere: intraterrestrial life on earth. *Annu. Rev. Earth Planet. Sci.* 40 551–568. 10.1146/annurev-earth-042711-105500

[B60] Edwards K. J. Fisher A. T. Wheat C. G. (2012b). the deep subsurface biosphere in igneous ocean crust: frontier habitats for microbiological exploration. *Front. Microbiol.* 3:8. 10.3389/fmicb.2012.00008 22347212 PMC3271274

[B61] Ehlmann B. L. Mustard J. F. Murchie S. L. (2010). Geologic setting of serpentine deposits on Mars. *Geophys. Res. Lett.* 37:L06201 10.1029/2010GL042596

[B62] Emerson D. Rentz J. A. Lilburn T. G. Davis R. E. Aldrich H. C. Chan C. S. (2007). A novel lineage of proteobacteria involved in formation of marine Fe-oxidizing microbial mat communities. *PLoS One* 2:e667. 10.1371/journal.pone.0000667 17668050 PMC1930151

[B63] Engelen B. Ziegelmüller K. Wolf L. Köpke B. Gittel A. Cypionka H. (2008). Fluids from the oceanic crust support microbial activities within the deep biosphere. *Geomicrobiol. J.* 25 56–66. 10.1080/01490450701829006

[B64] Escudero L. V. Casamayor E. O. Chong G. Pedrós-Alió C. Demergasso C. (2013). Distribution of microbial arsenic reduction, oxidation and extrusion genes along a wide range of environmental arsenic concentrations. *PLoS One* 8:e78890. 10.1371/journal.pone.0078890 24205341 PMC3815024

[B65] Fairén A. G. Davila A. F. Lim D. S. S. Bramall N. Bonaccorsi R. Zavaleta J. (2010). Astrobiology through the ages of mars: the study of terrestrial analogues to understand the habitability of mars. *Astrobiology* 10 821–843. 10.1089/ast.2009.0440 21087162

[B66] Fairén A. G. Parro V. Schulze-Makuch D. Whyte L. (2017). Searching for life on mars before it is too late. *Astrobiology* 17 962–970. 10.1089/ast.2017.1703 28885042 PMC5655416

[B67] Faucher B. Lacelle D. Davila A. F. Pollard W. Fisher D. McKay C. P. (2017). Physicochemical and biological controls on carbon and nitrogen in permafrost from an ultraxerous environment, McMurdo Dry Valleys of Antarctica. *J. Geophys. Res. Biogeosci.* 122 2593–2604. 10.1002/2017JG004006

[B68] Fernández-Remolar D. C. Prieto-Ballesteros O. Rodríguez N. Gómez F. Amils R. Gómez-Elvira J. (2008). Underground Habitats in the Río Tinto Basin: a model for subsurface life habitats on mars. *Astrobiology* 8 1023–1047. 10.1089/ast.2006.0104 19105758

[B69] Fisher A. T. (2005). Marine hydrogeology: recent accomplishments and future opportunities. *Hydrogeol. J.* 13 69–97. 10.1007/s10040-004-0400-y

[B70] Fortes A. D. Grindrod P. M. Trickett S. K. Vočadlo L. (2007). Ammonium sulfate on Titan: possible origin and role in cryovolcanism. *Icarus* 188 139–153. 10.1016/j.icarus.2006.11.002

[B71] Foster S. S. D. Chilton P. J. (2003). Groundwater: the processes and global significance of aquifer degradation. *Philos. Trans. R. Soc. B Biol. Sci.* 358 1957–1972. 10.1098/rstb.2003.1380 14728791 PMC1693287

[B72] Fredrickson J. K. Balkwill D. L. (2006). Geomicrobial processes and biodiversity in the deep terrestrial subsurface. *Geomicrobiol. J.* 23 345–356. 10.1080/01490450600875571 26917241

[B73] Froelich P. N. Klinkhammer G. P. Bender M. L. Luedtke N. A. Heath G. R. Cullen D. (1979). Early oxidation of organic matter in pelagic sediments of the eastern equatorial Atlantic: suboxic diagenesis. *Geochim. Cosmochim. Acta* 43 1075–1090. 10.1016/0016-7037(79)90095-4

[B74] Furnes H. Staudigel H. (1999). Biological mediation in ocean crust alteration: how deep is the deep biosphere? *Earth Planet. Sci. Lett.* 166 97–103. 10.1016/S0012-821X(99)00005-9

[B75] Garcia-Lopez E. Cid C. (2017). Glaciers and ice sheets as analog environments of potentially habitable Icy worlds. *Front. Microbiol.* 8:1407. 10.3389/fmicb.2017.01407 28804477 PMC5532398

[B76] García-Moyano A. González-Toril E. Aguilera Á. Amils R. (2012). Comparative microbial ecology study of the sediments and the water column of the Río Tinto, an extreme acidic environment. *FEMS Microbiol. Ecol.* 81 303–314. 10.1111/j.1574-6941.2012.01346.x 22385317

[B77] German C. R. Bowen A. Coleman M. L. Honig D. L. Huber J. A. Jakuba M. V. (2010). Diverse styles of submarine venting on the ultraslow spreading Mid-Cayman Rise. *Proc. Natl. Acad. Sci. U.S.A.* 107 14020–14025. 10.1073/pnas.1009205107 20660317 PMC2922602

[B1000] Gleeson T. Befus K. M. Jasechko S. Luijendijk E. Cardenas M. B. (2016). The global volume and distribution of modern groundwater. *Nat. Geosci.* 9 161–164. 10.1038/ngeo2590

[B78] Glein C. R. Baross J. A. Waite J. H. (2015). The pH of Enceladus’ ocean. *Geochim. Cosmochim. Acta* 162 202–219. 10.1016/j.gca.2015.04.017 19553992

[B79] Glombitza C. Adhikari R. R. Riedinger N. Gilhooly W. P. Hinrichs K.-U. Inagaki F. (2016). Microbial sulfate reduction potential in coal-bearing sediments down to ∼2.5 km below the seafloor off Shimokita Peninsula, Japan. *Front. Microbiol.* 7:1576. 10.3389/fmicb.2016.01576 27761134 PMC5051215

[B80] Goordial J. Davila A. F. Lacelle D. Pollard W. Marinova M. M. Greer C. W. (2016). Nearing the cold-arid limits of microbial life in permafrost of an upper dry valley, Antarctica. *ISME J.* 10 1613–1624. 10.1038/ismej.2015.239 27323892 PMC4918446

[B81] Hand K. P. Carlson R. W. Chyba C. F. (2007). Energy, chemical disequilibrium, and geological constraints on Europa. *Astrobiology* 7 1006–1022. 10.1089/ast.2007.0156 18163875

[B82] Hatzenpichler R. Connon S. A. Goudeau D. Malmstrom R. R. Woyke T. Orphan V. J. (2016). Visualizing in situ translational activity for identifying and sorting slow-growing archaeal-bacterial consortia. *Proc. Natl. Acad. Sci. U.S.A.* 113 E4069–E4078. 10.1073/pnas.1603757113 27357680 PMC4948357

[B83] Hayes A. Aharonson O. Callahan P. Elachi C. Gim Y. Kirk R. L. (2008). Hydrocarbon lakes on Titan: distribution and interaction with a porous regolith. *Geophys. Res. Lett.* 35:L09204 10.1029/2008gl033409

[B84] Head I. M. Gray N. D. Larter S. R. (2014). Life in the slow lane; biogeochemistry of biodegraded petroleum containing reservoirs and implications for energy recovery and carbon management. *Front. Microbiol.* 5:566. 10.3389/fmicb.2014.00566 25426105 PMC4227522

[B85] Head I. M. Jones D. M. Larter S. R. (2003). Biological activity in the deep subsurface and the origin of heavy oil. *Nature* 426 344–352. 10.1038/nature02134 14628064

[B86] Hedrich S. Schlomann M. Johnson D. B. (2011). The iron-oxidizing proteobacteria. *Microbiology* 157 1551–1564. 10.1099/mic.0.045344-0 21511765

[B87] Heim C. Quéric N. V. Ionescu D. Schäfer N. Reitner J. (2017). Frutexites-like structures formed by iron oxidizing biofilms in the continental subsurface (Äspö Hard Rock Laboratory, Sweden). *PLoS One* 12:e0177542. 10.1371/journal.pone.0177542 28542238 PMC5438144

[B88] Heldmann J. L. Pollard W. McKay C. P. Marinova M. M. Davila A. F. Williams K. E. (2013). The high elevation Dry Valleys in Antarctica as analog sites for subsurface ice on Mars. *Planet. Space Sci.* 85 53–58. 10.1016/j.pss.2013.05.019

[B89] Hernsdorf A. W. Amano Y. Miyakawa K. Ise K. Suzuki Y. Anantharaman K. (2017). Potential for microbial H2 and metal transformations associated with novel bacteria and archaea in deep terrestrial subsurface sediments. *ISME J.* 11 1915–1929. 10.1038/ismej.2017.39 28350393 PMC5520028

[B90] Hoefen T. M. Clark R. N. Bandfield J. L. Smith M. D. Pearl J. C. Christensen P. R. (2003). Discovery of olivine in the Nili Fssae region of Mars. *Science* 302 627–630. 10.1126/science.1089647 14576430

[B91] Hoehler T. M. (2004). Biological energy requirements as quantitative boundary conditions for life in the subsurface. *Geobiology* 2 205–215. 10.1111/j.1472-4677.2004.00033.x

[B92] Hoehler T. M. (2007). An Energy Balance Concept for Habitability. *Astrobiology* 7 824–838. 10.1089/ast.2006.0095 18163865

[B93] Hoehler T. M. Jørgensen B. B. (2013). Microbial life under extreme energy limitation. *Nat. Rev. Microbiol.* 11 83–94. 10.1038/nrmicro2939 23321532

[B94] Holm N. G. Oze C. Mousis O. Waite J. H. Guilbert-Lepoutre A. (2015). Serpentinization and the Formation of H_2_ and CH_4_ on Celestial Bodies (Planets, Moons, Comets). *Astrobiology* 15 587–600. 10.1089/ast.2014.1188 26154779 PMC4523005

[B95] Horgan B. H. N. Smith R. J. Cloutis E. A. Mann P. Christensen P. R. (2017). Acid weathering of basalt and basaltic glass: I. Near-infrared spectra, thermal-infrared spectra, and implications for Mars. *J. Geophys. Res. Planets* 122 1–31. 10.1002/2016JE005111

[B96] Hoshino T. Inagaki F. (2017). Distribution of anaerobic carbon monoxide dehydrogenase genes in deep subseafloor sediments. *Lett. Appl. Microbiol.* 64 355–363. 10.1111/lam.12727 28256106

[B97] Hsu H.-W. Postberg F. Sekine Y. Shibuya T. Kempf S. Horanyi M. (2015). Ongoing hydrothermal activities within Enceladus. *Nature* 519 207–210. 10.1038/nature14262 25762281

[B98] Iess L. Stevenson D. J. Parisi M. Hemingway D. Jacobson R. A. Lunine J. I. (2014). The gravity field and interior structure of Enceladus. *Science* 344 78–80. 10.1126/science.1250551 24700854

[B99] Jaakkola S. T. Ravantti J. J. Oksanen H. M. Bamford D. H. (2016). Buried alive: microbes from ancient halite. *Trends Microbiol.* 24 148–160. 10.1016/j.tim.2015.12.002 26796472

[B100] Jackson A. Davila A. F. Böhlke J. K. Sturchio N. C. Sevanthi R. Estrada N. (2016). Deposition, accumulation, and alteration of Cl -, NO 3 -, ClO 4 - and ClO 3 - salts in a hyper-arid polar environment: Mass balance and isotopic constraints. *Geochim. Cosmochim. Acta* 182 197–215. 10.1016/j.gca.2016.03.012

[B101] Jakosky B. M. Nealson K. H. Bakermans C. Ley R. E. Mellon M. T. (2003). Subfreezing activity of microorganisms and the potential habitability of mars’, polar regions. *Astrobiology* 3 343–350. 10.1089/153110703769016433 14577883

[B102] Jennings D. E. Flasar F. M. Kunde V. G. Samuelson R. E. Pearl J. C. Nixon C. A. (2009). Titan’s surface brightness temperatures. *Astrophys. J.* 691 L103–L105. 10.1088/0004-637X/691/2/L103

[B103] Ji F. Zhou H. Yang Q. Gao H. Wang H. Lilley M. D. (2017). Geochemistry of hydrothermal vent fluids and its implications for subsurface processes at the active Longqi hydrothermal field, Southwest Indian Ridge. *Deep Sea Res. Part 1 Oceanogr. Res. Pap.* 122 41–47. 10.1016/j.dsr.2017.02.001

[B104] Johnson D. B. Hedrich S. Pakostova E. (2017). Indirect redox transformations of iron, copper, and chromium catalyzed by extremely acidophilic bacteria. *Front. Microbiol.* 8:211. 10.3389/fmicb.2017.00211 28239375 PMC5301019

[B105] Johnson D. B. Kanao T. Hedrich S. (2012). Redox transformations of iron at extremely low pH: fundamental and applied aspects. *Front. Microbiol.* 3:96. 10.3389/fmicb.2012.00096 22438853 PMC3305923

[B106] Johnson S. S. Hebsgaard M. B. Christensen T. R. Mastepanov M. Nielsen R. Munch K. (2007). Ancient bacteria show evidence of DNA repair. *Proc. Natl. Acad. Sci. U.S.A.* 104 14401–14405. 10.1073/pnas.0706787104 17728401 PMC1958816

[B107] Jones S. E. Lennon J. T. (2010). Dormancy contributes to the maintenance of microbial diversity. *Proc. Natl. Acad. Sci. U.S.A.* 107 5881–5886. 10.1073/pnas.0912765107 20231463 PMC2851880

[B108] Jørgensen B. B. Boetius A. (2007). Feast and famine — microbial life in the deep-sea bed. *Nat. Rev. Microbiol.* 5 770–781. 10.1038/nrmicro1745 17828281

[B109] Jørgensen S. L. Zhao R. (2016). Microbial inventory of deeply buried oceanic crust from a young ridge flank. *Front. Microbiol.* 7:820. 10.3389/fmicb.2016.00820 27303398 PMC4882963

[B110] Joye S. B. Bowles M. W. Samarkin V. A. Hunter K. S. Niemann H. (2010). Biogeochemical signatures and microbial activity of different cold-seep habitats along the Gulf of Mexico deep slope. *Deep. Res. Pt. II Top. Stud. Oceanogr.* 57 1990–2001. 10.1016/j.dsr2.2010.06.001

[B111] Joye S. B. Samarkin V. A. Orcutt B. N. MacDonald I. R. Hinrichs K.-U. Elvert M. (2009). Metabolic variability in seafloor brines revealed by carbon and sulphur dynamics. *Nat. Geosci.* 2 349–354. 10.1038/ngeo475

[B112] Jungbluth S. P. Bowers R. M. Lin H. T. Cowen J. P. Rappé M. S. (2016). Novel microbial assemblages inhabiting crustal fluids within mid-ocean ridge flank subsurface basalt. *ISME J.* 10 2033–2047. 10.1038/ismej.2015.248 26872042 PMC5029167

[B113] Kallmeyer J. Pockalny R. Adhikari R. R. Smith D. C. D’Hondt S. (2012). Global distribution of microbial abundance and biomass in subseafloor sediment. *Proc. Natl. Acad. Sci. U.S.A.* 109 16213–16216. 10.1073/pnas.1203849109 22927371 PMC3479597

[B114] Kappler A. Emerson D. Edwards K. J. Amend J. P. Gralnick J. A. Grathwohl P. (2005). Microbial activity in biogeochemical gradients - New aspects of research. *Geobiology* 3 229–233. 10.1111/j.1472-4669.2005.00053.x

[B115] Kargel J. S. (1994). Cryovolcanism on the icy satellites. *Earth Moon Planets* 67 101–113. 10.1007/BF00613296

[B116] Karson J. A. (2002). Geologic structure of the uppermost oceanic crust created at fast- to intermediate-rate spreading centers. *Annu. Rev. Earth Planet. Sci.* 30 347–384. 10.1146/annurev.earth.30.091201.141132

[B117] Kashefi K. Lovley D. R. (2000). Reduction of Fe (III), Mn (IV), and Toxic Metals at 100°C by *Pyrobaculum islandicum* Reduction of Fe (III), Mn (IV), and Toxic Metals at 100°C by *Pyrobaculum islandicum*. *Society* 66 1050–1056. 10.1128/AEM.66.3.1050-1056.2000 PMC91941 10698770

[B118] Katayama T. Yoshioka H. Takahashi H. A. Amo M. Fujii T. Sakata S. (2016). Changes in microbial communities associated with gas hydrates in subseafloor sediments from the Nankai Trough. *FEMS Microbiol. Ecol.* 92:fiw093. 10.1093/femsec/fiw093 27170363

[B119] Kelley D. S. Karson J. A. Blackman D. K. Früh-Green G. L. Butterfield D. A. Lilley M. D. (2001). An off-axis hydrothermal vent field near the Mid-Atlantic Ridge at 30°N. *Nature* 412 145–149. 10.1038/35084000 11449263

[B120] Kelley D. S. Karson J. A. Früh-Green G. L. Yoerger D. R. Shank T. M. Butterfield D. A. (2005). A serpentinite-hosted ecosystem: the Lost City hydrothermal field. *Science* 307 1428–1434. 10.1126/science.1102556 15746419

[B121] Kempes C. P. van Bodegom P. M. Wolpert D. Libby E. Amend J. P. Hoehler T. M. (2017). Drivers of bacterial maintenance and minimal energy requirements. *Front. Microbiol.* 8:31. 10.3389/fmicb.2017.00031 28197128 PMC5281582

[B122] Kieffer S. W. Lu X. Bethke C. M. Spencer J. R. Marshak S. Navrotsky A. (2006). A clathrate reservoir hypothesis for enceladus south polar plume. *Science* 314 1764–1766. 10.1126/science.1133519 17170301

[B123] Kivelson M. G. Khurana K. K. Russell C. T. Volwerk M. Walker R. J. Zimmer C. (2000). Galileo magnetometer measurements: a stronger case for a subsurface ocean at Europa. *Science* 289 1340–1343. 10.1126/science.289.5483.1340 10958778

[B124] Koh C. A. (2002). Towards a fundamental understanding of natural gas hydrates. *Chem. Soc. Rev.* 31 157–167. 10.1039/b008672j 12122641

[B125] Kounaves S. P. Hecht M. H. Kapit J. Gospodinova K. DeFlores L. Quinn R. C. (2010a). Wet chemistry experiments on the 2007 phoenix mars scout lander mission: data analysis and results. *J. Geophys. Res.* 115:E00E10 10.1029/2009JE003424

[B126] Kounaves S. P. Stroble S. T. Anderson R. M. Moore Q. Catling D. C. Douglas S. (2010b). Discovery of natural perchlorate in the Antarctic dry valleys and its global implications. *Environ. Sci. Technol.* 44 2360–2364. 10.1021/es9033606 20155929

[B127] Kracke F. Vassilev I. Kromer J. O. (2015). Microbial electron transport and energy conservation - The foundation for optimizing bioelectrochemical systems. *Front. Microbiol.* 6:575. 10.3389/fmicb.2015.00575 26124754 PMC4463002

[B128] Kristjánsson J. K. Hreggvidsson G. O. (1995). Ecology and habitats of extremophiles. *World J. Microbiol. Biotechnol.* 11 17–25. 10.1007/BF00339134 24414409

[B129] Labonté J. M. Lever M. A. Edwards K. J. Orcutt B. N. (2017). Influence of igneous basement on deep sediment microbial diversity on the eastern Juan de Fuca Ridge flank. *Front. Microbiol.* 8:1434. 10.3389/fmicb.2017.01434 28824568 PMC5539551

[B130] Lang S. Q. Butterfield D. A. Schulte M. Kelley D. S. Lilley M. D. (2010). Elevated concentrations of formate, acetate and dissolved organic carbon found at the Lost City hydrothermal field. *Geochim. Cosmochim. Acta* 74 941–952. 10.1016/j.gca.2009.10.045

[B131] Lang S. Q. Früh-Green G. L. Bernasconi S. M. Brazelton W. J. Schrenk M. O. McGonigle J. M. (2018). Deeply-sourced formate fuels sulfate reducers but not methanogens at Lost City hydrothermal field. *Sci. Rep.* 8:755. 10.1038/s41598-017-19002-5 29335466 PMC5768773

[B132] LaRowe D. E. Amend J. P. (2015a). Catabolic rates, population sizes and doubling/replacement times of microorganisms in natural settings. *Am. J. Sci.* 315 167–203. 10.2475/03.2015.01

[B133] LaRowe D. E. Amend J. P. (2015b). Power limits for microbial life. *Front. Microbiol.* 6:718 10.3389/fmicb.2015.00718 PMC4502533 26236299

[B134] LaRowe D. E. Burwicz E. Arndt S. Dale A. W. Amend J. P. (2017). Temperature and volume of global marine sediments. *Geology* 45 275–278. 10.1130/G38601.1

[B135] LaRowe D. E. Van Cappellen P. (2011). Degradation of natural organic matter: a thermodynamic analysis. *Geochim. Cosmochim. Acta* 75 2030–2042. 10.1016/j.gca.2011.01.020

[B136] Larter S. R. Huang H. Adams J. Bennett B. Jokanola O. Oldenburg T. (2006). The controls on the composition of biodegraded oils in the deep subsurface: part II - Geological controls on subsurface biodegradation fluxes and constraints on reservoir-fluid property prediction. *Am. Assoc. Pet. Geol. Bull.* 90 921–938. 10.1306/01270605130

[B137] Lau M. C. Y. Kieft T. L. Kuloyo O. Linage-Alvarez B. van Heerden E. Lindsay M. R. (2016). An oligotrophic deep-subsurface community dependent on syntrophy is dominated by sulfur-driven autotrophic denitrifiers. *Proc. Natl. Acad. Sci. U.S.A.* 113 E7927–E7936. 10.1073/pnas.1612244113 27872277 PMC5150411

[B138] Lederberg J. (1960). Exobiology: approaches to life beyond the Earth. *Science* 132 393–400. 14415126 10.1126/science.132.3424.393

[B139] Lee J.-H. Fredrickson J. K. Plymale A. E. Dohnalkova A. C. Resch C. T. McKinley J. P. (2015). An autotrophic H_2_ -oxidizing, nitrate-respiring, Tc(VII)-reducing A Cidovorax sp. isolated from a subsurface oxic-anoxic transition zone. *Environ. Microbiol. Rep.* 7 395–403. 10.1111/1758-2229.12263 25558059

[B140] Lehman R. M. Roberto F. F. Earley D. Bruhn D. F. Brink S. E. O’Connell S. P. (2001). Attached and unattached bacterial communities in a 120-Meter Corehole in an Acidic, Crystalline Rock Aquifer. *Appl. Environ. Microbiol.* 67 2095–2106. 10.1128/AEM.67.5.2095-2106.2001 11319087 PMC92842

[B141] Lennon J. T. Jones S. E. (2011). Microbial seed banks: the ecological and evolutionary implications of dormancy. *Nat. Rev. Microbiol.* 9 119–130. 10.1038/nrmicro2504 21233850

[B142] Leupin O. X. Bernier-Latmani R. Bagnoud A. Moors H. Leys N. Wouters K. (2017). Fifteen years of microbiological investigation in Opalinus Clay at the Mont Terri rock laboratory (Switzerland). *Swiss J. Geosci.* 110 343–354. 10.1007/s00015-016-0255-y PMC7081829 32214982

[B143] Léveillé R. (2010). A half-century of terrestrial analog studies: from craters on the Moon to searching for life on Mars. *Planet. Space Sci.* 58 631–638. 10.1016/j.pss.2009.04.001

[B144] Lever M. A. Rogers K. L. Lloyd K. G. Overmann J. Schink B. Thauer R. K. (2015). Life under extreme energy limitation: a synthesis of laboratory- and field-based investigations. *FEMS Microbiol. Rev.* 39 688–728. 10.1093/femsre/fuv020 25994609

[B145] L’Haridon S. Reysenbacht A.-L. Glénat P. Prieur D. Jeanthon C. (1995). Hot subterranean biosphere in a continental oil reservoir. *Nature* 377 223–224. 10.1038/377223a0 8588738

[B146] Lopes R. M. C. Kirk R. L. Mitchell K. L. Legall A. Barnes J. W. Hayes A. (2013). Cryovolcanism on Titan: new results from Cassini RADAR and VIMS. *J. Geophys. Res. E Planets* 118 416–435. 10.1002/jgre.20062

[B147] Lovley D. R. (2008). Extracellular electron transfer: Wires, capacitors, iron lungs, and more. *Geobiology* 6 225–231. 10.1111/j.1472-4669.2008.00148.x 18393985

[B148] Lovley D. R. Chapelle F. H. (1995). Deep subsurface microbial processes. *Rev. Geophys.* 33 365–381. 10.1029/95RG01305

[B149] Lowell R. P. DuBose M. (2005). Hydrothermal systems on Europa. *Geophys. Res. Lett.* 32:L05202 10.1029/2005gl022375

[B150] Lowenstein T. K. Schubert B. A. Timofeeff M. N. (2011). Microbial communities in fluid inclusions and long-term survival in halite. *GSA Today* 21 4–9. 10.1130/GSATG81A.1 29350297

[B151] Lunine J. I. (2017). Ocean worlds exploration. *Acta Astronaut.* 131 123–130. 10.1016/j.actaastro.2016.11.017

[B152] Lysnes K. Thorseth I. H. Steinsbu B. O. Øvreås L. Torsvik T. Pedersen R. B. (2004). Microbial community diversity in seafloor basalt from the Arctic spreading ridges. *FEMS Microbiol. Ecol.* 50 213–230. 10.1016/j.femsec.2004.06.014 19712362

[B153] Marlow J. J. Skennerton C. T. Li Z. Chourey K. Hettich R. L. Pan C. (2016). Proteomic stable isotope probing reveals biosynthesis dynamics of slow growing methane based microbial communities. *Front. Microbiol.* 7:563. 10.3389/fmicb.2016.00563 27199908 PMC4850331

[B154] Martin A. McMinn A. (2017). Sea ice, extremophiles and life on extra-terrestrial ocean worlds. *Int. J. Astrobiol.* 17 1–16. 10.1017/s1473550416000483

[B155] Martins Z. Cottin H. Kotler J. M. Carrasco N. Cockell C. S. Dela Torre (2017). Earth as a tool for astrobiology—a European perspective. *Space Sci. Rev.* 209 43–81. 10.1007/s11214-017-0369-1

[B156] McCollom T. M. (1999). Methanogenesis as a potential source of chemical energy for primary biomass production by autotrophic organisms in hydrothermal systems on Europa. *J. Geophys. Res. Planets* 104 30729–30742. 10.1029/1999JE001126

[B157] McCord T. B. Hansen G. B. Fanale F. P. Carlson R. W. Matson D. L. Johnson T. V. (1998). Salts on Europa’s surface detected by Galileo’s near infrared mapping spectrometer. *Science* 280 1242–1245. 10.1126/science.280.5367.1242 9596573

[B158] McMahon S. Parnell J. (2014). Weighing the deep continental biosphere. *FEMS Microbiol. Ecol.* 87 113–120. 10.1111/1574-6941.12196 23991863

[B159] Meyer J. L. Jaekel U. Tully B. J. Glazer B. T. Wheat C. G. Lin H.-T. (2016). A distinct and active bacterial community in cold oxygenated fluids circulating beneath the western flank of the Mid-Atlantic ridge. *Sci. Rep.* 6:22541. 10.1038/srep22541 26935537 PMC4776111

[B160] Michaud A. B. Dore J. E. Achberger A. M. Christner B. C. Mitchell A. C. Skidmore M. L. (2017). Microbial oxidation as a methane sink beneath the West Antarctic Ice Sheet. *Nat. Geosci.* 10 582–586. 10.1038/ngeo2992

[B161] Mickol R. L. Kral T. A. (2017). Low pressure tolerance by methanogens in an aqueous environment: implications for subsurface life on Mars. *Orig. Life Evol. Biosph.* 47 511–532. 10.1007/s11084-016-9519-9 27663448

[B162] Miettinen H. Kietäväinen R. Sohlberg E. Numminen M. Ahonen L. Itävaara M. (2015). Microbiome composition and geochemical characteristics of deep subsurface high-pressure environment, Pyhasalmi mine Finland. *Front. Microbiol.* 6:1203. 10.3389/fmicb.2015.01203 26579109 PMC4626562

[B163] Mikucki J. A. Lee P. A. Ghosh D. Purcell A. M. Mitchell A. C. Mankoff K. D. (2016). Subglacial Lake Whillans microbial biogeochemistry: a synthesis of current knowledge. *Philos. Trans. A Math. Phys. Eng. Sci.* 374:20140290. 10.1098/rsta.2014.0290 26667908

[B164] Momper L. M. Kiel Reese B. Zinke L. A. Wanger G. Osburn M. R. Moser D. P. (2017). Major phylum-level differences between porefluid and host rock bacterial communities in the terrestrial deep subsurface. *Environ. Microbiol. Rep.* 9 501–511. 10.1111/1758-2229.12563 28677247

[B165] Morono Y. Terada T. Nishizawa M. Ito M. Hillion F. Takahata N. (2011). Carbon and nitrogen assimilation in deep subseafloor microbial cells. *Proc. Natl. Acad. Sci. U.S.A.* 108 18295–18300. 10.1073/pnas.1107763108 21987801 PMC3215001

[B166] Mousis O. Choukroun M. Lunine J. I. Sotin C. (2014). Equilibrium composition between liquid and clathrate reservoirs on Titan. *Icarus* 239 39–45. 10.1016/j.icarus.2014.05.032 25774974

[B167] Müller V. Hess V. (2017). The minimum biological energy quantum. *Front. Microbiol.* 8:2019 10.3389/fmicb.2017.02019 PMC5662883 29123504

[B168] Mumma M. J. Villanueva G. L. Novak R. E. Hewagama T. Bonev B. P. DiSanti M. A. (2009). Strong release of methane on Mars in northern summer 2003. *Science* 323 1041–1045. 10.1126/science.1165243 19150811

[B169] Murray A. E. Kenig F. Fritsen C. H. McKay C. P. Cawley K. M. Edwards R. (2012). Microbial life at -13 C in the brine of an ice-sealed Antarctic lake. *Proc. Natl. Acad. Sci. U.S.A.* 109 20626–20631. 10.1073/pnas.1208607109 23185006 PMC3528574

[B170] Mykytczuk N. C. S. Foote S. J. Omelon C. R. Southam G. Greer C. W. Whyte L. G. (2013). Bacterial growth at - 15°C; molecular insights from the permafrost bacterium *Planococcus halocryophilus* Or1. *ISME J.* 7 1211–1226. 10.1038/ismej.2013.8 23389107 PMC3660685

[B171] Navarro-González R. Rainey F. A. Molina P. Bagaley D. R. Hollen B. J. De La Rosa J. (2003). Mars-like soils in the Atacama desert, chile, and the dry limit of microbial life. *Science* 302 1018–1021. 10.1126/science.1089143 14605363

[B172] Navarro-González R. Vargas E. De La Rosa J. Raga A. C. McKay C. P. (2010). Reanalysis of the Viking results suggests perchlorate and organics at midlatitudes on Mars. *J. Geophys. Res. E Planets* 115:E12010 10.1029/2010JE003599

[B173] Neubeck A. Sun L. Müller B. Ivarsson M. Hosgörmez H. Özcan D. (2017). Microbial community structure in a serpentine-hosted abiotic gas seepage at the Chimaera Ophiolite, Turkey. *Appl. Environ. Microbiol.* 83:e03430-16. 10.1128/AEM.03430-16 28389534 PMC5452829

[B174] Niemann H. B. Atreya S. K. Bauer S. H. Carignan G. R. Demick J. E. Frost R. L. (2005). The abundances of constituents of Titan’s atmosphere from the GCMS instrument on the Huygens probe. *Nature* 438 779–784. 10.1038/nature04122 16319830

[B175] Nixon S. L. Cockell C. S. Tranter M. (2012). Limitations to a microbial iron cycle on Mars. *Planet. Space Sci.* 72 116–128. 10.1016/j.pss.2012.04.003

[B176] Nixon S. L. Cousins C. R. Cockell C. S. (2013). Plausible microbial metabolisms on Mars. *Astron. Geophys.* 54 13–11. 10.1093/astrogeo/ats034 29966361

[B177] Ody A. Poulet F. Langevin Y. Bibring J. P. Bellucci G. Altieri F. (2012). Global maps of anhydrous minerals at the surface of Mars from OMEGA/MEx. *J. Geophys. Res. E Planets* 117: E00J14 10.1029/2012JE004117

[B178] Oehler D. Z. Etiope G. (2017). Methane seepage on Mars: where to look and why. *Astrobiology* 17 1233–1264. 10.1089/ast.2017.1657 28771029 PMC5730060

[B179] Oger P. M. Jebbar M. (2010). The many ways of coping with pressure. *Res. Microbiol.* 161 799–809. 10.1016/j.resmic.2010.09.017 21035541

[B180] Orcutt B. N. Edwards K. J. (2014). Life in the ocean crust: lessons from subseafloor laboratories. *Dev. Mar. Geol.* 7 175–196. 10.1016/B978-0-444-62617-2.00007-4

[B181] Orcutt B. N. Larowe D. E. Biddle J. F. Colwell F. S. Glazer B. T. Reese B. K. (2013a). Microbial activity in the marine deep biosphere: progress and prospects. *Front. Microbiol.* 4:189. 10.3389/fmicb.2013.00189 23874326 PMC3708129

[B182] Orcutt B. N. Wheat C. G. Rouxel O. J. Hulme S. M. Edwards K. J. Bach W. (2013b). Oxygen consumption rates in subseafloor basaltic crust derived from a reaction transport model. *Nat. Commun.* 4:2539. 10.1038/ncomms3539 24071791

[B183] Orcutt B. N. Sylvan J. B. Knab N. J. Edwards K. J. (2011). Microbial ecology of the dark ocean above, at, and below the seafloor. *Microbiol. Mol. Biol. Rev.* 75 361–422. 10.1128/MMBR.00039-10 21646433 PMC3122624

[B184] Orcutt B. N. Sylvan J. B. Rogers D. R. Delaney J. Lee R. W. Girguis P. R. (2015). Carbon fixation by basalt-hosted microbial communities. *Front. Microbiol.* 6:904. 10.3389/fmicb.2015.00904 26441854 PMC4561358

[B185] Oremland R. S. (2003). The ecology of Arsenic. *Science* 300 939–944. 10.1126/science.1081903 12738852

[B186] Orsi W. D. Barker Jørgensen B. Biddle J. F. (2016). Transcriptional analysis of sulfate reducing and chemolithoautotrophic sulfur oxidizing bacteria in the deep subseafloor. *Environ. Microbiol. Rep.* 8 452–460. 10.1111/1758-2229.12387 26991974

[B187] Orsi W. D. Edgcomb V. P. Christman G. D. Biddle J. F. (2013). Gene expression in the deep biosphere. *Nature* 499 205–208. 10.1038/nature12230 23760485

[B188] Osburn M. R. LaRowe D. E. Momper L. M. Amend J. P. (2014). Chemolithotrophy in the continental deep subsurface: Sanford Underground Research Facility (SURF), USA. *Front. Microbiol.* 5:610. 10.3389/fmicb.2014.00610 25429287 PMC4228859

[B189] Osegovic J. P. Max M. D. (2005). Compound clathrate hydrate on Titan’s surface. *J. Geophys. Res. E Planets* 110:E08004 10.1029/2005JE002435

[B190] Panikov N. S. Sizova M. V. (2007). Growth kinetics of microorganisms isolated from Alaskan soil and permafrost in solid media frozen down to −35 degrees C. *FEMS Microbiol. Ecol.* 59 500–512. 10.1111/j.1574-6941.2006.00210.x 17026514

[B191] Parnell J. McMahon S. (2016). Physical and chemical controls on habitats for life in the deep subsurface beneath continents and ice. *Philos. Trans. R. Soc. A Math. Phys. Eng. Sci.* 374:20140293. 10.1098/rsta.2014.0293 26667907 PMC4685966

[B192] Parro V. de Diego-Castilla G. Moreno-Paz M. Blanco Y. Cruz-Gil P. Rodríguez-Manfredi J. A. (2011). A microbial oasis in the hypersaline Atacama subsurface discovered by a life detector chip: implications for the search for life on mars. *Astrobiology* 11 969–996. 10.1089/ast.2011.0654 22149750 PMC3242637

[B193] Payler S. J. Biddle J. F. Coates A. J. Cousins C. R. Cross R. E. Cullen D. C. (2017). Planetary science and exploration in the deep subsurface: results from the MINAR Program, Boulby Mine, UK. *Int. J. Astrobiol.* 16 114–129. 10.1017/S1473550416000045

[B194] Peng X. Ta K. Chen S. Zhang L. Xu H. (2015). Coexistence of Fe(II)- and Mn(II)-oxidizing bacteria govern the formation of deep sea umber deposits. *Geochim. Cosmochim. Acta* 169 200–216. 10.1016/j.gca.2015.09.011

[B195] Peretyazhko T. S. Niles P. B. Sutter B. Morris R. V. Agresti D. G. Le L. (2017). Smectite formation in the presence of sulfuric acid: implications for acidic smectite formation on early Mars. *Geochim. Cosmochim. Acta* 220 248–260. 10.1016/j.gca.2017.10.004 PMC7427815 32801388

[B196] Phelps T. J. Murphy E. M. Pfiffner S. M. White D. C. (1994). Comparison between geochemical and biological estimates of subsurface microbial activities. *Microb. Ecol.* 28 335–349. 10.1007/BF00662027 24186553

[B197] Postberg F. Kempf S. Schmidt J. Brilliantov N. Beinsen A. Abel B. (2009). Sodium salts in E-ring ice grains from an ocean below the surface of Enceladus. *Nature* 459 1098–1101. 10.1038/nature08046 19553992

[B198] Preston L. J. Dartnell L. R. (2014). Planetary habitability: lessons learned from terrestrial analogues. *Int. J. Astrobiol.* 13 81–98. 10.1017/S1473550413000396

[B199] Price P. B. Sowers T. (2004). Temperature dependence of metabolic rates for microbial growth, maintenance, and survival. *Proc. Natl. Acad. Sci. U.S.A.* 101 4631–4636. 10.1073/pnas.0400522101 15070769 PMC384798

[B200] Prokofeva M. I. Kublanov I. V. Nercessian O. Tourova T. P. Kolganova T. V. Lebedinsky A. V. (2005). Cultivated anaerobic acidophilic/acidotolerant thermophiles from terrestrial and deep-sea hydrothermal habitats. *Extremophiles* 9 437–448. 10.1007/s00792-005-0461-4 15970992

[B201] Proskurowski G. Lilley M. D. Seewald J. S. Früh-Green G. L. Olson E. J. Lupton J. E. (2008). Abiogenic hydrocarbon production at lost city hydrothermal field. *Science* 319 604–607. 10.1126/science.1151194 18239121

[B202] Purkamo L. Bomberg M. Nyyssönen M. Ahonen L. Kukkonen I. Itävaara M. (2017). Response of deep subsurface microbial community to different carbon sources and electron acceptors during 2 months incubation in microcosms. *Front. Microbiol.* 8:232. 10.3389/fmicb.2017.00232 28265265 PMC5316538

[B203] Quick L. C. Marsh B. D. (2016). Heat transfer of ascending cryomagma on Europa. *J. Volcanol. Geotherm. Res.* 319 66–77. 10.1016/j.jvolgeores.2016.03.018

[B204] Reese B. K. Zinke L. A. Sobol M. S. LaRowe D. E. Orcutt B. N. Zhang X. (2018). Nitrogen cycling of active bacteria within oligotrophic sediment of the mid-atlantic ridge flank. *Geomicrobiol. J.* 35 468–483. 10.1080/01490451.2017.1392649

[B205] Rempfert K. R. Miller H. M. Bompard N. Nothaft D. Matter J. M. Kelemen P. (2017). Geological and geochemical controls on subsurface microbial life in the Samail Ophiolite, Oman. *Front. Microbiol.* 8:56. 10.3389/fmicb.2017.00056 28223966 PMC5293757

[B206] Reveillaud J. Reddington E. McDermott J. Algar C. Meyer J. L. Sylva S. (2016). Subseafloor microbial communities in hydrogen-rich vent fluids from hydrothermal systems along the Mid-Cayman Rise. *Environ. Microbiol.* 18 1970–1987. 10.1111/1462-2920.13173 26663423 PMC5021209

[B207] Reynolds G. T. Lutz R. A. (2001). Sources of Light in the Deep Ocean. *Rev. Geophys.* 39 123–136. 10.1029/1999RG000071

[B208] Robador A. LaRowe D. E. Jungbluth S. P. Lin H. T. Rappé M. S. Nealson K. H. (2016a). Nanocalorimetric characterization of microbial activity in deep subsurface oceanic crustal fluids. *Front. Microbiol.* 7:454. 10.3389/fmicb.2016.00454 27092118 PMC4820435

[B209] Robador A. Müller A. L. Sawicka J. E. Berry D. Hubert C. R. J. Loy A. (2016b). Activity and community structures of sulfate-reducing microorganisms in polar, temperate and tropical marine sediments. *ISME J.* 10 796–809. 10.1038/ismej.2015.157 26359912 PMC4796921

[B210] Røy H. Kallmeyer J. Adhikari R. R. Pockalny R. Jørgensen B. B. D’Hondt S. (2012). Aerobic microbial respiration in 86-million-year-old deep-sea red clay. *Science* 336 922–925. 10.1126/science.1219424 22605778

[B211] Rudnick R. L. Fountain D. M. (1995). Nature and composition of the continental crust: a lower crustal perspective. *Rev. Geophys.* 33 267–309. 10.1029/95RG01302

[B212] Rudnick R. L. Gao S. (2003). 3.01 – Composition of the continental crust. *Treatise Geochem.* 3 1–64. 10.1016/b0-08-043751-6/03016-4

[B213] Sánchez-Andrea I. Knittel K. Amann R. I. Amils R. Sanz J. L. (2012). Quantification of Tinto river sediment microbial communities: importance of sulfate-reducing bacteria and their role in attenuating acid mine drainage. *Appl. Environ. Microbiol.* 78 4638–4645. 10.1128/AEM.00848-12 22544246 PMC3370487

[B214] Sánchez-Andrea I. Rodríguez N. Amils R. Sanz J. L. (2011). Microbial diversity in anaerobic sediments at Río Tinto, a naturally acidic environment with a high heavy metal content. *Appl. Environ. Microbiol.* 77 6085–6093. 10.1128/AEM.00654-11 21724883 PMC3165421

[B215] Schink B. (1997). Energetics of syntrophic cooperation in methanogenic degradation. *Microbiol. Mol. Biol. Rev.* 61 262–280. 9184013 10.1128/mmbr.61.2.262-280.1997 PMC232610

[B216] Schink B. Stams A. (2006). Syntrophism among prokaryotes. *Prokaryotes* 2 309–335.

[B217] Schmidt B. E. Blankenship D. D. Patterson G. W. Schenk P. M. (2011). Active formation of “chaos terrain” over shallow subsurface water on Europa. *Nature* 479 502–505. 10.1038/nature10608 22089135

[B218] Schrenk M. O. Brazelton W. J. Lang S. Q. (2013). Serpentinization, carbon, and deep life. *Rev. Mineral. Geochem.* 75 575–606. 10.2138/rmg.2013.75.18

[B219] Schulte M. Blake D. Hoehler T. M. McCollom T. M. (2006). Serpentinization and It’s Implication for Life on Early Earth and Mars. *Astrobiology* 6 364–376. 16689652 10.1089/ast.2006.6.364

[B220] Sekine Y. Shibuya T. Postberg F. Hsu H. W. Suzuki K. Masaki Y. (2015). High-temperature water-rock interactions and hydrothermal environments in the chondrite-like core of Enceladus. *Nat. Commun.* 6:8604. 10.1038/ncomms9604 26506464 PMC4639802

[B221] Siegert M. J. Ross N. Le Brocq A. M. (2016). Recent advances in understanding Antarctic subglacial lakes and hydrology. *Philos. Trans. R. Soc. A Math. Phys. Eng. Sci.* 374:20140306. 10.1098/rsta.2014.0306 26667914 PMC4685968

[B222] Skidmore M. L. Foght J. M. Sharp M. J. (2000). Microbial life beneath a high Arctic glacier. *Appl. Environ. Microbiol.* 66 3214–3220. 10.1128/AEM.66.8.3214-3220.2000 10919772 PMC92136

[B223] Slobodkin A. I. Slobodkina G. B. (2014). Thermophilic prokaryotes from deep subterranean habitats. *Microbiology* 83 169–183. 10.1134/S0026261714030151 25844436

[B224] Smith J. A. Tremblay P. L. Shrestha P. M. Snoeyenbos-West O. L. Franks A. E. Nevin K. P. (2014). Going wireless: Fe(III) oxide reduction without pili by Geobacter sulfurreducens strain JS-1. *Appl. Environ. Microbiol.* 80 4331–4340. 10.1128/AEM.01122-14 24814783 PMC4068678

[B225] Spilde M. N. Northup D. E. Boston P. J. Schelble R. T. Dano K. E. Crossey L. J. (2005). Geomicrobiology of cave ferromanganese deposits: a field and laboratory investigation. *Geomicrobiol. J.* 22 99–116. 10.1080/01490450590945889

[B226] Squyres S. W. Grotzinger J. P. Arvidson R. E. Bell J. F. Calvin W. Christensen P. R. (2004). *In situ* evidence for an ancient aqueous environment at Meridiani Planum, Mars. *Science* 306 1709–1714. 10.1126/science.1104559 15576604

[B227] Staudigel H. Yayanos A. Chastain R. Davies G. Verdurmen E. A. Schiffman P. (1998). Biologically mediated dissolution of volcanic glass in seawater. *Earth Planet. Sci. Lett.* 164 233–244. 10.1016/S0012-821X(98)00207-6

[B228] Stern J. C. Sutter B. Freissinet C. Navarro-González R. McKay C. P. Archer P. D. (2015). Evidence for indigenous nitrogen in sedimentary and aeolian deposits from the Curiosity rover investigations at Gale crater, Mars. *Proc. Natl. Acad. Sci. U.S.A.* 112 4245–4250. 10.1073/pnas.1420932112 25831544 PMC4394254

[B229] Stevens T. Mckinley J. P. (1995). Lithoautotrophic microbial ecosystems in deep basalt aquifers. *Science* 270 450–454. 10.1038/s41467-017-01288-8 29051484 PMC5648843

[B230] Stober I. Bucher K. (2004). Fluid sinks within the earth’s crust. *Geofluids* 4 143–151. 10.1111/j.1468-8115.2004.00078.x

[B231] Stofan E. R. Elachi C. Lunine J. I. Lorenz R. D. Stiles B. Mitchell K. L. (2007). The lakes of Titan. *Nature* 445 61–64. 10.1038/nature05438 17203056

[B2000] Sylvan J. B. Hoffman C. L. Momper L. M. Toner B. M. Amend J. P. Edwards K. J. (2015). *Bacillus rigiliprofundi* sp. nov., an endospore-forming Mn-oxidizing, moderately halophilic bacterium isolated from deep subseafloor basaltic crust. *Int. J. Syst. Evol. Microbiol.* 65 1992–1998. 10.1099/ijs.0.000211 25813363

[B232] Szponar N. Brazelton W. J. Schrenk M. O. Bower D. M. Steele A. Morrill P. L. (2013). Geochemistry of a continental site of serpentinization, the Tablelands Ophiolite, Gros Morne National Park: A Mars analogue. *Icarus* 224 286–296. 10.1016/j.icarus.2012.07.004

[B233] Takai K. Nakamura K. Toki T. Tsunogai U. Miyazaki M. Miyazaki J. (2008). Cell proliferation at 122 C and isotopically heavy CH4 production by a hyperthermophilic methanogen under high-pressure cultivation. *Proc. Natl. Acad. Sci. U.S.A.* 105 10949–10954. 10.1073/pnas.0712334105 18664583 PMC2490668

[B234] Teal L. R. Bulling M. T. Parker E. R. Solan M. (2008). Global patterns of bioturbation intensity and mixed depth of marine soft sediments. *Aquat. Biol.* 2 207–218. 10.3354/ab00052

[B235] Teolis B. D. Plainaki C. Cassidy T. A. Raut U. (2017). Water Ice Radiolytic O_2_, H_2_, and H_2_O_2_ yields for any projectile species, energy, or temperature: a model for icy astrophysical bodies. *J. Geophys. Res. Planets* 122 1996–2012. 10.1002/2017JE005285

[B236] Toner B. M. Rouxel O. J. Santelli C. M. Bach W. Edwards K. J. (2016). Iron transformation pathways and redox micro-environments in seafloor sulfide-mineral deposits: Spatially resolved Fe XAS and δ57/54Fe observations. *Front. Microbiol.* 7:648. 10.3389/fmicb.2016.00648 27242685 PMC4862312

[B237] Trembath-Reichert E. Morono Y. Ijiri A. Hoshino T. Dawson K. S. Inagaki F. (2017). Methyl-compound use and slow growth characterize microbial life in 2-km-deep subseafloor coal and shale beds. *Proc. Natl. Acad. Sci. U.S.A.* 114 E9206–E9215. 10.1073/pnas.1707525114 29078310 PMC5676895

[B238] Tsesmetzis N. Alsop E. B. Vigneron A. Marcelis F. Head I. M. Lomans B. P. (2016). Microbial community analysis of three hydrocarbon reservoir cores provides valuable insights for the assessment of reservoir souring potential. *Int. Biodeterior. Biodegrad.* 126 177–188.

[B239] Tully B. J. Wheat C. G. Glazer B. T. Huber J. A. (2018). A dynamic microbial community with high functional redundancy inhabits the cold, oxic subseafloor aquifer. *ISME J.* 12 1–16. 10.1038/ismej.2017.187 29099490 PMC5739024

[B240] Van Dover C. L. Reynolds G. T. Chave A. D. Tyson J. A. (1996). Light at deep-sea hydrothermal vents. *Geophys. Res. Lett.* 23 2049–2052. 10.1029/96GL02151

[B241] Vance S. D. Hand K. P. Pappalardo R. T. (2016). Geophysical controls of chemical disequilibria in Europa. *Geophys. Res. Lett.* 43 4871–4879. 10.1002/2016GL068547

[B242] Volpi M. Lomstein B. A. Sichert A. Røy H. Jørgensen B. B. Kjeldsen K. U. (2017). Identity, abundance, and reactivation kinetics of thermophilic fermentative endospores in cold marine sediment and seawater. *Front. Microbiol.* 8:131. 10.3389/fmicb.2017.00131 28220111 PMC5292427

[B243] Waite J. H. Lewis W. S. Magee B. A. Lunine J. I. McKinnon W. B. Glein C. R. (2009). Liquid water on Enceladus from observations of ammonia and40Ar in the plume. *Nature* 460 487–490. 10.1038/nature08153

[B244] Webster C. R. Mahaffy P. R. Atreya S. K. Flesch G. J. Mischna M. A. Meslin P.-Y. (2015). Mars methane detection and variability at Gale crater. *Science* 347 415–417. 10.1126/science.1261713 25515120

[B245] Wedepohl K. H. (1995). The composition of the continental crust. *Geochim. Cosmochim. Acta* 59 1217–1232. 10.1016/0016-7037(95)00038-2

[B246] Wegener G. Bausch M. Holler T. Thang N. M. Prieto Mollar X. Kellermann M. Y. (2012). Assessing sub-seafloor microbial activity by combined stable isotope probing with deuterated water and 13C-bicarbonate. *Environ. Microbiol.* 14 1517–1527. 10.1111/j.1462-2920.2012.02739.x 22498240

[B247] Weiss B. P. Yung Y. L. Nealson K. H. (2000). Atmospheric energy for subsurface life on Mars? *Proc. Natl. Acad. Sci. U.S.A.* 97 1395–1399. 10.1073/pnas.030538097 10660689 PMC26444

[B248] White S. N. Chave A. D. Reynolds G. T. (2002). Investigations of ambient light emission at deep-sea hydrothermal vents. *J. Geophys. Res. Solid Earth* 107 EPM 1-1–EPM 1-13. 10.1029/2000JB000015

[B249] Whiticar M. J. (1990). A geochemial perspective of natural gas and atmospheric methane. *Org. Geochem.* 16 531–547. 10.1016/0146-6380(90)90068-B

[B250] Whitman W. B. Coleman D. C. Wiebe W. J. (1998). Prokaryotes: the unseen majority. *Proc. Natl. Acad. Sci. U.S.A.* 95 6578–6583. 10.1073/pnas.95.12.6578 9618454 PMC33863

[B251] Wilhelms A. Larter S. R. Head I. M. Farrimond P. Di-Primio R. Zwach C. (2001). Biodegradation of oil in uplifted basins prevented by deep-burial sterilization. *Nature* 411 1034–1037. 10.1038/35082535 11429600

[B252] Williams P. J. Cloete T. E. (2008). Microbial community study of the iron ore concentrate of the Sishen Iron Ore Mine, South Africa. *World J. Microbiol. Biotechnol.* 24 2531–2538. 10.1007/s11274-008-9777-4

[B253] Wirsen C. O. Jannasch H. W. (1978). Physiological and morphological observations on *Thiovulum* sp. *J. Bacteriol.* 136 765–774. 101531 10.1128/jb.136.2.765-774.1978 PMC218603

[B254] Wu X. Holmfeldt K. Hubalek V. Lundin D. Åström M. Bertilsson S. (2016). Microbial metagenomes from three aquifers in the Fennoscandian shield terrestrial deep biosphere reveal metabolic partitioning among populations. *ISME J.* 10 1192–1203. 10.1038/ismej.2015.185 26484735 PMC5029217

[B255] Zhang X. Feng X. Wang F. (2016). Diversity and metabolic potentials of subsurface crustal microorganisms from the western flank of the mid-atlantic ridge. *Front. Microbiol.* 7:363. 10.3389/fmicb.2016.00363 27047476 PMC4797314

[B256] Ziebis W. McManus J. Ferdelman T. G. Schmidt-Schierhorn F. Bach W. Muratli J. (2012). Interstitial fluid chemistry of sediments underlying the North Atlantic gyre and the influence of subsurface fluid flow. *Earth Planet. Sci. Lett.* 32 79–91. 10.1016/j.epsl.2012.01.018

