# Corrigendum: Low Energy Subsurface Environments as Extraterrestrial Analogs

**DOI:** 10.3389/fmicb.2019.01959

**Published:** 2019-08-27

**Authors:** Rose M. Jones, Jacqueline M. Goordial, Beth N. Orcutt

**Affiliations:** Bigelow Laboratory for Ocean Sciences, East Boothbay, ME, United States

**Keywords:** deep biosphere, subsurface, astrobiology, low energy, energy limitation

In the original article, there was a mistake in the calculations in **Supplementary Table S1** as published, which affected Figure 4 and the resulting text describing Figure 4.

**FIGURE 4:**
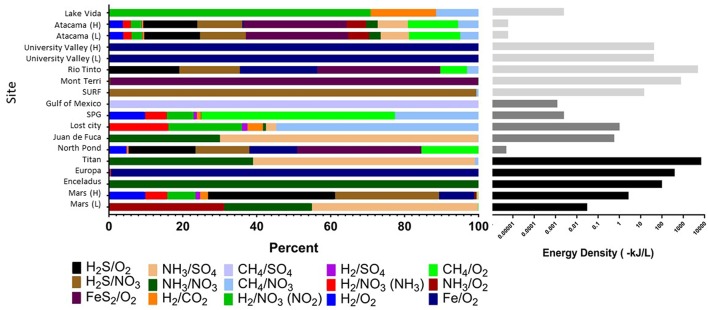
Cumulative volumetric energy densities of redox reactions per site based on environmental concentrations of variables in each reaction (Table 1). **(Left)** Shows the percent contribution of various reactions as shown in the color legend. **(Right)** Shows combined absolute energy density (as kJ per liter) for all reactions, with gray scale reflecting habitat type. All calculations assumed that the electron donor was the limiting substrate. See Supplementary Table S1 for all values and formulas.

In brief, there were errors in the equations for calculating the volumetric energy densities for some of the reactions, due to a copy/paste error, which affected the estimates of cumulative energy density and ratio of reactions presented in Figure 4. We have corrected these errors in the revised **Supplementary Table S1** file as well as in Figure 4, shown below. We thank the reader who brought this to our attention.

**Supplementary Table S1**: The file has been corrected in the original article.

Due to the error mentioned above, a correction has been made to section ***Energetics and*** ***the Subsurface***, subsection ***Energy Yield of Various Redox Reactions in the Low*** ***Energy Subsurface and on Extraterrestrial Environments***, paragraphs four and five:

“Strikingly, extraterrestrial sites are predicted to have similar cumulative energy densities as Earth's subsurface habitats (with conservative assumptions about electron donor and acceptor concentrations), although the dominant energy-rich processes vary (Figure 4, Supplementary Table S1). For example, cumulative volumetric energy densities on Mars are estimated to range from 0.03 to 3 kJ L^−1^, supported primarily by the electron donors NH_3_, H_2_S, or hydrogen reacting with sulfate, nitrate, or oxygen, depending on the scenario chosen for electron donor concentration, pH, and temperature. Under the scenario of low electron donor concentration, low pH, and low temperature, the predicted Martian energy density and dominant reactions are similar to those observed at the Earth analog site at the Juan de Fuca Ridge flank subsurface oceanic crust. Under the scenario of higher electron donor concentrations, pH, and temperature, the cumulative volumetric energy density and dominant reactions estimate is more similar to what is estimated from the Earth analog sites in the Rio Tinto. The base of the presumed Europan ocean has an estimated energy density of 400 kJ L^−1^ fueled primarily by iron oxidation, if dissolved oxygen is present (Teolis et al., 2017) and penetrates to the water-rock interface and if iron is released from water-rock reactions. This volumetric energy density and dominant reaction pattern is similar to that estimated for the Earth analog site at University Valley. By contrast, the ocean on Enceladus is estimated to have an energy density of 100 kJ L^−1^ fueled by ammonia oxidation with nitrate; none of our comparison Earth analog sites had similar energy density estimates from this reaction. The cumulative volumetric energy density estimates for Titan are the highest we estimate in this exercise, fueled by ammonia oxidation with sulfate or nitrate in a similar pattern as estimated for the Juan de Fuca analog system, but we highlight that this is the least well constrained system. Overall, although based on poorly constrained concentrations, these projections indicate that extraterrestrial sites could have sufficient overall energy to host chemolithotrophic communities.”

“The predicted relative contribution of each redox pair to each site is applicable information for the “follow the energy” approach to habitability (Hoehler, 2007), and can further be constrained by comparison studies of microbial metabolic processes in the Earth analog systems, to see if the predicted energy rich metabolisms are indeed those that occur. This approach of comparing energy density to microbial community function has recently been shown for some subsurface sites (Osburn et al., 2014; Reveillaud et al., 2016; Momper et al., 2017), demonstrating the power of this energy density approach to be a useful predictor of metabolic function. For example, North Pond energy is primarily from the FeS_2_/O_2_ couple (Figure 4), indicating that solid mineral substrates may be significant in this environment. Oxidation of hydrogen sulfide is also predicted to yield more energy than other electron donors (Figure 4), which agrees well with information on metabolic function in the community indicating that sulfur oxidizers are present in greater relative abundance as compared to hydrogen, ammonia and nitrite metabolisms (Jørgensen and Zhao, 2016; Meyer et al., 2016). Lost City estimates show methane and hydrogen oxidation reactions as significant sources of energy (Figure 4), which agrees with work indicating methane oxidizers are common in this system but contrasts with other recent work pointing to sulfate metabolisms as being more important than hydrogen metabolisms in this environment (Lang et al., 2018). At this site, the Gibbs free energy of the H_2_/CO_2_ couple is relatively high but the energy density low (Figures 3, 4), as dissolved CO_2_ concentration is scarce because it rapidly precipitates as carbonates in the high pH environment. As shown previously, sulfide oxidizing metabolisms are energy rich in the continental subsurface at the Sanford Underground Research Facility, and sulfide oxidizers are dominant in the microbial community (Osburn et al., 2014; Momper et al., 2017). In the subsurface portion of Rio Tinto, observation of iron and sulfur metabolisms matches with estimates of energy density (García-Moyano et al., 2012; Sánchez-Andrea et al., 2012; Amils et al., 2014). The Atacama analog site has a very low predicted energy availability, although we note that factors like water availability may be more important than energy availability in structuring the microbial community at the hyperarid and polar desert environments (Goordial et al., 2016). It is notable that the range of pH and temperature scenarios at the Atacama and University Valley sites did not particularly affect the predicted dominant reactions or volumetric energy densities at the hyper-arid sites, unlike the Mars sites, which notably changed, highlighting that the ion concentrations are key for determining dominant reactions and energy densities. Overall, this “follow the energy” approach of matching predicting energy density to microbial community structure and function may inform the likely metabolisms that might be found on extraterrestrial targets.”

In addition, in the original article, the following references were incorrectly written.

The reference for “Amend et al., 2015” should be “Sylvan, J. B., Hoffman, C. L., Momper, L. M., Toner, B. M., Amend, J. P., and Edwards, K. J. (2015). *Bacillus rigiliprofundi* sp. nov., an endospore-forming Mn-oxidizing, moderately halophilic bacterium isolated from deep seubseafloor basaltic crust. *Int. J. Syst. Evol. Microbiol*. 65, 1992–1998. doi: 10.1099/ijs.0.000211.” The citation in the text has been updated accordingly.

The reference for “Amils et al., 2014” should be “Amils, R., Fernández-Remolar, D. C., and IPBSL Team (2014). Río Tinto: a geochemical and mineralogical terrestrial analogue of mars. *Life* 4, 511–534. doi: 10.3390/life4030511.”

The reference for “Bate et al., 2004” should be “Gleeson, T., Befus, K. M., Jasechko, S., Luijendijk, E., and Cardenas, M. B. (2016). The global volume and distribution of modern groundwater. *Nat. Geosci*. 9, 161–164. doi: 10.1038/ngeo2590.” The citation in the text has been updated accordingly.

The reference for “Lowell, 2005” should be “Lowell, R. P., and DuBose M. (2005). Hydrothermal systems on Europa. *Geophys. Res. Lett*. 32:L05202. doi: 10.1029/2005gl022375.”

The reference for “McCord, 1998” should be “McCord, T. B., Hansen, G. B., Fanale, F. P., Carlson, R.W., Matson, D. L., Johnson, T. V., et al. (1998). Salts on Europa's surface detected by Galileo's near infrared mapping spectrometer. *Science* 280, 1242–1245. doi: 10.1126/science.280.5367.1242.”

The reference for “Schink et al., 2006” should be “Schink, B., and Stams, A. (2006). Syntrophism among prokaryotes. *Prokaryotes* 2, 309–335.”

The reference for “Squyres, 2004” should be “Squyres, S. W., Grotzinger, J. P., Arvidson, R. E., Bell, J. F., Calvin, W., Christensen, P. R., et al. (2004). *In situ* evidence for an ancient aqueous environment at Meridiani Planum, Mars. *Science* 306, 1709–1714. doi: 10.1126/science.1104559.”

The authors apologize for these errors and state that they do not change the scientific conclusions of the article in any way. The original article has been updated.
