# Supplementary material for: Low Energy Subsurface Environments as Extraterrestrial Analogs
Source: Front Microbiol. 2018 Jul 18;9:1605. doi: 10.3389/fmicb.2018.01605 (PMC6058055; doi:10.3389/fmicb.2018.01605)
Supplement: TABLE S2 — Source references for values in Supplementary Table S1. [file Table_2.DOCX]

| Site | # | References |
| --- | --- | --- |
| **Extraterrestrial** |  |  |
| Mars | 1 | (Dehouck et al., 2012; Kounaves et al., 2010a, 2010b; Morris et al., 2000; Nixon et al., 2013; Stern et al., 2015; Webster et al., 2015) |
|  | 2 |  |
| Enceladus | 3 | (Bach and Edwards, 2003; Glein et al., 2015; Marion et al., 2012; Postberg et al., 2009; Waite et al., 2006, 2009) |
| Europa | 4 | (Bach and Edwards, 2003; Carlson et al., 1999; Kargel et al., 2000; Lowell, 2005; Melosh et al., 2004; Schmidt et al., 2011; Vance et al., 2016; Woodland et al., 2006) |
| Titan | 5 | (Brassé et al., 2017; Cordier et al., 2009, 2013; de Kok et al., 2007; Marion et al., 2012; Vuitton et al., 2006) |
| **Marine** |  |  |
| North Pond | 6 | (Meyer et al., 2016) |
| Juan de Fuca | 7 | (Mehta et al., 2003) |
| Lost City | 8 | (Kelley et al., 2005) |
| South Pacific Gyre (SPG) | 9 | (D’Hondt, 2004; D’Hondt et al., 2003) |
| Gulf of Mexico | 10 | (Bowles et al., 2016; Joye et al., 2010; Roberts et al., 2010) |
| **Continental** |  |  |
| Sanford Underground Research Facility (SURF) | 11 | (Osburn et al., 2014) |
| Mont Terri | 12 | (Bagnoud et al., 2016; Bossart and Milnes; Pearson et al., 2003) |
| Rio Tinto | 13 | (García-Moyano et al., 2007; Sánchez-Andrea et al., 2012) |
| University Valley | 14 | (Gunn, 1962; Kounaves et al., 2010c) |
|  | 15 |  |
| Atacama | 16 | (Crits-Christoph et al., 2013; McKay et al., 2003; Navarro-González et al., 2003) |
|  | 17 |  |
| Lake Vida | 18 | (Murray et al., 2012) |

Reference:

Bach, W., and Edwards, K. J. (2003). Iron and sulfide oxidation within the basaltic ocean crust: Implications for chemolithoautotrophic microbial biomass production. *Geochim. Cosmochim. Acta* 67, 3871–3887. doi:10.1016/S0016-7037(03)00304-1.

Bagnoud, A., de Bruijn, I., Andersson, A. F., Diomidis, N., Leupin, O. X., Schwyn, B., et al. (2016). A minimalistic microbial food web in an excavated deep subsurface clay rock. *FEMS Microbiol. Ecol.* 92, fiv138. doi:10.1093/femsec/fiv138.

Bossart, P., and Milnes, A. G. *Mont Terri Rock Laboratory, 20 Years : Two Decades of Research and Experimentation on Claystones for Geological Disposal of Radioactive Waste*. Available at: https://books.google.com/books?id=xgxEDwAAQBAJ&dq=opalinus+clay+methane&source=gbs_navlinks_s [Accessed January 23, 2018].

Bowles, M. W., Hunter, K. S., Samarkin, V. A., and Joye, S. B. (2016). Patterns and variability in geochemical signatures and microbial activity within and between diverse cold seep habitats along the lower continental slope, Northern Gulf of Mexico. *Deep Sea Res. Part II Top. Stud. Oceanogr.* 129, 31–40. doi:10.1016/j.dsr2.2016.02.011.

Brassé, C., Buch, A., Coll, P., and Raulin, F. (2017). Low-Temperature Alkaline pH Hydrolysis of Oxygen-Free Titan Tholins: Carbonates’ Impact. *Astrobiology* 17, 8–26. doi:10.1089/ast.2016.1524.

Carlson, R. W., Johnson, R. E., and Anderson, M. S. (1999). Sulfuric Acid on Europa and the Radiolytic Sulfur Cycle. *Science (80-. ).* 286, 97–99. doi:10.1126/science.286.5437.97.

Cordier, D., Mousis, O., Lunine, J. I., Lavvas, P., and Vuitton, V. (2009). An estimate of the chemical composition of titan’s lakes. *Astrophys. J.* 707. doi:10.1088/0004-637X/707/2/L128.

Cordier, D., Mousis, O., Lunine, J. I., Lavvas, P., and Vuitton, V. (2013). Erratum: An estimate of the chemical composition of titan’s lakes (Astrophysical Journal Letters (2009) 707 (L128)). *Astrophys. J. Lett.* 768, L23. doi:10.1088/2041-8205/768/1/L23.

Crits-Christoph, A., Robinson, C. K., Barnum, T. P., Fricke, W., Davila, A. F., Jedynak, B., et al. (2013). Colonization patterns of soil microbial communities in the Atacama Desert. *Microbiome* 1, 28. doi:10.1186/2049-2618-1-28.

D’Hondt, S. (2004). Distributions of Microbial Activities in Deep Subseafloor Sediments. *Science (80-. ).* 306, 2216–2221. doi:10.1126/science.1101155.

D’Hondt, S. L., Jørgensen, B. B., and Miller, D. J. eds. (2003). *Proceedings of the Ocean Drilling Program, 201 Initial Reports*. Ocean Drilling Program doi:10.2973/odp.proc.ir.201.2003.

de Kok, R., Irwin, P. G. J., Teanby, N. A., Lellouch, E., Bézard, B., Vinatier, S., et al. (2007). Oxygen compounds in Titan’s stratosphere as observed by Cassini CIRS. *Icarus* 186, 354–363. doi:10.1016/j.icarus.2006.09.016.

Dehouck, E., Chevrier, V. F., Gaudin, A., Mangold, N., Mathé, P.-E., and Rochette, P. (2012). Evaluating the role of sulfide-weathering in the formation of sulfates or carbonates on Mars. *Geochim. Cosmochim. Acta* 90, 47–63. doi:10.1016/j.gca.2012.04.057.

García-Moyano, A., González-Toril, E., Aguilera, Á., and Amils, R. (2007). Prokaryotic community composition and ecology of floating macroscopic filaments from an extreme acidic environment, Río Tinto (SW, Spain). *Syst. Appl. Microbiol.* 30, 601–614. doi:10.1016/j.syapm.2007.08.002.

Glein, C. R., Baross, J. A., and Waite, J. H. (2015). The pH of Enceladus’ ocean. *Geochim. Cosmochim. Acta* 162, 202–219. doi:10.1016/j.gca.2015.04.017.

Gunn, B. M. (1962). Differentiation in Ferrar dolerites, Antarctica. *New Zeal. J. Geol. Geophys.* 5, 820–863. doi:10.1080/00288306.1962.10417641.

Joye, S. B., Bowles, M. W., Samarkin, V. A., Hunter, K. S., and Niemann, H. (2010). Biogeochemical signatures and microbial activity of different cold-seep habitats along the Gulf of Mexico deep slope. *Deep. Res. Part II Top. Stud. Oceanogr.* 57, 1990–2001. doi:10.1016/j.dsr2.2010.06.001.

Kargel, J. S., Kaye, J. Z., Head, J. W., Marion, G. M., Sassen, R., Crowley, J. K., et al. (2000). Europa’s Crust and Ocean: Origin, Composition, and the Prospects for Life. *Icarus* 148, 226–265. doi:10.1006/icar.2000.6471.

Kelley, D. S., Karson, J. A., Früh-Green, G. L., Yoerger, D. R., Shank, T. M., Butterfield, D. A., et al. (2005). A serpentinite-hosted ecosystem: The Lost City hydrothermal field. *Science (80-. ).* 307, 1428–1434. doi:10.1126/science.1102556.

Kounaves, S. P., Hecht, M. H., Kapit, J., Gospodinova, K., DeFlores, L., Quinn, R. C., et al. (2010a). Wet Chemistry experiments on the 2007 Phoenix Mars Scout Lander mission: Data analysis and results. *J. Geophys. Res.* 115, E00E10. doi:10.1029/2009JE003424.

Kounaves, S. P., Hecht, M. H., Kapit, J., Quinn, R. C., Catling, D. C., Clark, B. C., et al. (2010b). Soluble sulfate in the martian soil at the Phoenix landing site. *Geophys. Res. Lett.* 37, n/a-n/a. doi:10.1029/2010GL042613.

Kounaves, S. P., Stroble, S. T., Anderson, R. M., Moore, Q., Catling, D. C., Douglas, S., et al. (2010c). Discovery of Natural Perchlorate in the Antarctic Dry Valleys and Its Global Implications. *Environ. Sci. Technol.* 44, 2360–2364. doi:10.1021/es9033606.

Lowell, R. P. (2005). Hydrothermal systems on Europa. *Geophys. Res. Lett.* 32. doi:10.1029/2005gl022375.

Marion, G. M., Kargel, J. S., Catling, D. C., and Lunine, J. I. (2012). Modeling ammonia–ammonium aqueous chemistries in the Solar System’s icy bodies. *Icarus* 220, 932–946. doi:10.1016/j.icarus.2012.06.016.

McKay, C. P., Friedmann, E. I., Gómez-Silva, B., Cáceres-Villanueva, L., Andersen, D. T., and Landheim, R. (2003). Temperature and moisture conditions for life in the extreme arid region of the Atacama desert: four years of observations including the El Niño of 1997-1998. *Astrobiology* 3, 393–406. doi:10.1089/153110703769016460.

Mehta, M. P., Butterfield, D. A., and Baross, J. A. (2003). Phylogenetic Diversity of Nitrogenase (nifH) Genes in Deep-Sea and Hydrothermal Vent Environments of the Juan de Fuca Ridge. *Appl. Environ. Microbiol.* 69, 960–970. doi:10.1128/AEM.69.2.960-970.2003.

Melosh, H. J., Ekholm, A. G., Showman, A. P., and Lorenz, R. D. (2004). The temperature of Europa’s subsurface water ocean. *Icarus* 168, 498–502. doi:10.1016/j.icarus.2003.11.026.

Meyer, J. L., Jaekel, U., Tully, B. J., Glazer, B. T., Wheat, C. G., Lin, H. T., et al. (2016). A distinct and active bacterial community in cold oxygenated fluids circulating beneath the western flank of the Mid-Atlantic ridge. *Sci. Rep.* 6, 22541. doi:10.1038/srep22541.

Morris, R. V., Golden, D. C., Bell, J. F., Shelfer, T. D., Scheinost, A. C., Hinman, N. W., et al. (2000). Mineralogy, composition, and alteration of Mars Pathfinder rocks and soils: Evidence from multispectral, elemental, and magnetic data on terrestrial analogue, SNC meteorite, and Pathfinder samples. *J. Geophys. Res. Planets* 105, 1757–1817. doi:10.1029/1999JE001059.

Murray, A. E., Kenig, F., Fritsen, C. H., McKay, C. P., Cawley, K. M., Edwards, R., et al. (2012). Microbial life at -13 C in the brine of an ice-sealed Antarctic lake. *Proc. Natl. Acad. Sci.* 109, 20626–20631. doi:10.1073/pnas.1208607109.

Navarro-González, R., Rainey, F. A., Molina, P., Bagaley, D. R., Hollen, B. J., De La Rosa, J., et al. (2003). Mars-Like Soils in the Atacama Desert, Chile, and the Dry Limit of Microbial Life. *Science (80-. ).* 302, 1018–1021. doi:10.1126/science.1089143.

Nixon, S. L., Cousins, C. R., and Cockell, C. S. (2013). Plausible microbial metabolisms on Mars. *Astron. Geophys.* 54, 1.13-1.16. doi:10.1093/astrogeo/ats034.

Osburn, M. R., LaRowe, D. E., Momper, L. M., and Amend, J. P. (2014). Chemolithotrophy in the continental deep subsurface: Sanford Underground Research Facility (SURF), USA. *Front. Microbiol.* 5, 610. doi:10.3389/fmicb.2014.00610.

Pearson, F. J., Arcos, D., Bath, A., Boisson, J., Fernández, A. M., Gäbler, H. E., et al. (2003). *Mont Terri Project – Geochemistry of Water in the Opalinus Clay Formation at the Mont Terri Rock Laboratory*.

Postberg, F., Kempf, S., Schmidt, J., Brilliantov, N., Beinsen, A., Abel, B., et al. (2009). Sodium salts in E-ring ice grains from an ocean below the surface of Enceladus. *Nature* 459, 1098–1101. doi:10.1038/nature08046.

Roberts, H. H., Feng, D., and Joye, S. B. (2010). Cold-seep carbonates of the middle and lower continental slope, northern Gulf of Mexico. *Deep. Res. Part II Top. Stud. Oceanogr.* 57, 2040–2054. doi:10.1016/j.dsr2.2010.09.003.

Sánchez-Andrea, I., Knittel, K., Amann, R. I., Amils, R., and Sanz, J. L. (2012). Quantification of Tinto river sediment microbial communities: Importance of sulfate-reducing bacteria and their role in attenuating acid mine drainage. *Appl. Environ. Microbiol.* 78, 4638–4645. doi:10.1128/AEM.00848-12.

Schmidt, B. E., Blankenship, D. D., Patterson, G. W., and Schenk, P. M. (2011). Active formation of “chaos terrain” over shallow subsurface water on Europa. *Nature* 479, 502–505. doi:10.1038/nature10608.

Stern, J. C., Sutter, B., Freissinet, C., Navarro-González, R., McKay, C. P., Archer, P. D., et al. (2015). Evidence for indigenous nitrogen in sedimentary and aeolian deposits from the Curiosity rover investigations at Gale crater, Mars. *Proc. Natl. Acad. Sci.* 112, 4245–4250. doi:10.1073/pnas.1420932112.

Vance, S. D., Hand, K. P., and Pappalardo, R. T. (2016). Geophysical controls of chemical disequilibria in Europa. *Geophys. Res. Lett.* 43, 4871–4879. doi:10.1002/2016GL068547.

Vuitton, V., Yelle, R. V., and Anicich, V. G. (2006). The Nitrogen Chemistry of Titan’s Upper Atmosphere Revealed. *Astrophys. J.* 647, L175–L178. doi:10.1086/507467.

Waite, J. H., Combi, M. R., Ip, W. H., Cravens, T. E., McNutt, R. L., Kasprzak, W., et al. (2006). Cassini ion and neutral mass spectrometer: Enceladus plume composition and structure. *Science (80-. ).* 311, 1419–1422. doi:10.1126/science.1121290.

Waite, J. H., Lewis, W. S., Magee, B. A., Lunine, J. I., McKinnon, W. B., Glein, C. R., et al. (2009). Liquid water on Enceladus from observations of ammonia and40Ar in the plume. *Nature* 460, 487–490. doi:10.1038/nature08153.

Webster, C. R., Mahaffy, P. R., Atreya, S. K., Flesch, G. J., Mischna, M. A., Meslin, P.-Y., et al. (2015). Mars methane detection and variability at Gale crater. *Science (80-. ).* 347, 415–417.

Woodland, A. B., Kornprobst, J., and Tabit, A. (2006). Ferric iron in orogenic lherzolite massifs and controls of oxygen fugacity in the upper mantle. *Lithos* 89, 222–241. doi:10.1016/j.lithos.2005.12.014.
